# Influence of the Nature of the Cationic Residues on the Activity of the Antimicrobial Peptides of the Lynronne Family

**DOI:** 10.1002/psc.70146

**Published:** 2026-11-03

**Authors:** Alexis Reinbold, Maxime Gâchon, Jean‐François Cavalier, Laure Genet, Sophie Guillier, Mathilde Lescat, Muriel Serradeil‐Albalat, Linda B. Oyama, Cendrine Nicoletti, Sharon A. Huws, Nicolas Vidal, Fabiano Cavalcanti Fernandes, Andreia Gomes Martins, Stéphane Canaan, Octávio Luiz Franco, Sophie Tronnet, Olga Iranzo, Marc Maresca

**Affiliations:** ^1^ Aix Marseille Univ, CNRS, Centrale Med, iSm2 Marseille France; ^2^ Unité Bactériologie, Département Microbiologie et Maladies Infectieuses, UMR‐MD1‐Inserm 1261 Institut de Recherche Biomédicale des Armées (IRBA) ‐ Brétigny‐Sur‐Orge France; ^3^ Aix‐Marseille Univ, CNRS, LISM UMR7255 Marseille France; ^4^ Aix Marseille Univ, CNRS, Centrale Med, FSCM Marseille France; ^5^ Institute for Global Food Security, School of Biological Sciences Queen's University Belfast Belfast UK; ^6^ Yelen Analytics Ensues La Redonne France; ^7^ Departamento de Ciência da Compuagomes@ação Instituto Federal de Brasília, Campus Taguatinga Brasília Brazil; ^8^ S‐Inova Biotech, Graduate Program in Biotechnology Dom Bosco Catholic University Campo Grande MS Brazil; ^9^ Center for Proteomic and Biochemical Analysis, Graduate Program in Genomic Sciences and Biotechnology Catholic University of Brasília Brasília DF Brazil

**Keywords:** AMP, antimicrobial peptides, biofilm, cationic residues, ESKAPEE, Lynronne

## Abstract

Most antimicrobial peptides (AMPs) are cationic, with positively charged amino acid residues (i.e., arginine, lysine, and/or histidine) being responsible for their selective interaction with bacteria. In the present study, we investigated the influence of the nature of cationic residues taking the Lynronnes peptides as models. Derivatives were designed and synthetized with substitutions of the type of cationic residues present. All derivatives and parental peptides were studied in terms of secondary structure, antimicrobial activity, anti‐biofilm activity, mechanism of action, and in vitro toxicity. All‐His peptides were found inactive, whereas all‐Lys variants showed the highest potency against Gram‐negative strains, particularly against *E. coli* . All‐Arg analogues were more active against Gram‐positive strains with enhanced stability under salt or serum conditions but were also more hemolytic. Mechanistic studies revealed that the peptides act by membrane permeabilization. Finally, Lynronne‐3 and its all‐Lys derivative proved the most effective in both the prevention and eradication of biofilms. These findings confirm that the type of cationic residue present in AMPs affects their activity, toxicity, and sensitivity to host factors, with different effects depending on the rest of the amino acid sequence, Lys‐substitution emerging as a promising strategy in the case of Lynronnes for targeting *E. coli* .

## Graphical abstract

Derivatives of Lynronnes with cationic residues substitution were designed and tested in terms of antimicrobial activity, anti‐biofilm activity, mechanism of action, and in vitro toxicity. Lys‐substitution emerges as a promising strategy for improved activity and selectivity, particularly against *E. coli* .

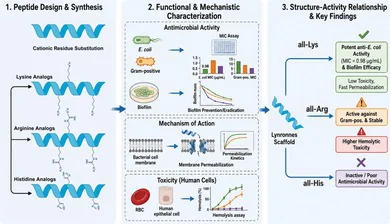

**Abbreviations**
AMPs: antimicrobial peptides
Arg: arginine
ATP: adenosine triphosphate
BSL‐2: Biosafety Level 2
BSL‐3: Biosafety Level 3
CC_50_
: cytotoxic concentration 50%
CD: circular dichroism
CFU: colony‐forming unit
DIPEA: *N*,*N*‐diisopropylethylamine
ESI‐MS: electrospray ionization mass spectrometry
Fmoc: fluorenylmethyloxycarbonyl
HATU: hexafluorophosphate azabenzotriazole tetramethyl uronium
HBTU: hexafluorophosphate benzotriazole tetramethyl uronium
HC_50_
: hemolytic concentration 50%
His: histidine
Lys: lysine
MBC: minimum bactericidal concentration
MBEC: minimal biofilm eradication concentration
MBIC: minimal biofilm inhibitory concentration
MH: Mueller–Hinton broth
MIC: minimum inhibitory concentration
OD_600_
: optical density 600 nm
RP‐HPLC: reversed‐phase high‐performance liquid chromatography
SDS: sodium dodecyl sulfate
SI: selectivity index
SPPS: solid‐phase peptide synthesis
tBu: tert‐butyl
UV: ultraviolet

## 1 Introduction

The history of antibiotics began in 1910 with Salvarsan [1] and accelerated with Alexander Fleming's discovery of penicillin in 1928 [2]. From the golden age (1940–1960) to the present day, more than 10,000 antibacterial molecules have revolutionized medicine, treating infections and supporting surgery and cancer treatment [3]. The main families are β‐lactams, tetracyclines, aminoglycosides, macrolides, and quinolones. These molecules are consumed by humans worldwide, though unevenly across regions. The largest share of total antibiotic use is in animals, representing approximately 70% of consumption [4, 5]. This overuse generates resistance through selective pressure, favoring survival and transmission of resistant variants [6]. At the same time, the number of new antibiotics discovered and marketed has steadily decreased in recent years, and the trend suggests this situation is unlikely to change [7]. A majority of resistant infections originate from a group of bacteria designated by the acronym ESKAPE ( *Enterococcus faecium* , *Staphylococcus aureus* , *Klebsiella pneumoniae* , *Acinetobacter baumannii* , *Pseudomonas aeruginosa* , and *Enterobacter* spp.), often extended to ESKAPEE to include *Escherichia coli* [8, 9]. This group, which comprises both Gram‐positive and Gram‐negative bacteria, is frequently encountered in hospitals and can cause nosocomial infections resistant to conventional antibiotics, potentially leading to fatalities in immunocompromised and intensive‐care patients [9, 10]. Importantly, once largely confined to hospital settings, infections caused by antibiotic resistant bacteria have increasingly emerged in the community especially in developing countries [11].

In addition to ESKAPEE bacteria, several bacterial species are responsible for severe human infections (such as mycobacteria) and are also of particular concern in the context of biological threats. Notably, *Bacillus anthracis* , *Burkholderia mallei* , *Burkholderia pseudomallei* , *Francisella tularensis* , and *Yersinia pestis* are classified as Tier 1 Select Agents in the United States [12]. The development of new antibacterial compounds active against these species is therefore of dual interest, both for the treatment of naturally occurring infections and for strengthening the availability of therapeutic alternatives in the context of biological threats. In this context, evaluating novel antimicrobial peptides (AMPs) against these bacterial species, together with *Brucella melitensis* [13], provides valuable information on their antibacterial spectrum and potential as alternative therapeutic agents [14].

In response to this challenge, AMPs have emerged as promising alternatives to antibiotics. Present in nearly all living organisms, they constitute a pillar of innate immunity, active against bacteria, fungi, parasites, and viruses [15, 16]. Their therapeutic potential dates back to 1922 with lysozyme, isolated by Fleming from tears and nasal secretions, a 130 amino‐acid antibacterial enzyme later eclipsed by penicillin [17]. The rise of resistance has brought AMPs back to the forefront, their multifactorial mechanisms (primarily membranolytic) limiting the emergence of resistance [18]. Despite the higher cost of peptide production compared with conventional antibiotics, the urgency of the situation is driving research in this field, with a surge in publications, alongside the fact that manufacturing costs are continuously decreasing [19]. Most AMPs range from 10 to 60 amino acids (average ~33), exhibit moderate hydrophobicity (~40%), and display amphipathic properties essential for membrane interaction, with an excess of basic residues including arginine (Arg), lysine (Lys), and histidine (His) [20, 21].

The cationic nature of AMPs (+2 to +9) dictates their electrostatic interaction with anionic bacterial surfaces (lipopolysaccharides (LPS), lipoteichoic acid (LTA), phosphatidylglycerol (PG), and cardiolipin (CL), causing a rapid bacteriolytic effect [22]. However, beyond the overall net charge, the chemical nature and distribution of basic side chains are known to be critical determinants of peptide‐membrane interactions and thus antimicrobial activity [23, 24]. Recent studies have highlighted that arginine and lysine, although carrying the same nominal charge, are not functionally interchangeable: The guanidinium moiety of arginine forms bidentate hydrogen bonds and interacts more strongly with lipid bilayers than the primary ammonium moiety of lysine; this difference can result in increased antibacterial activity but also in higher hemolysis or cytotoxicity, depending on the peptide scaffold considered and the bacteria [25]. Histidine offers a distinct mode of modulation, as protonation of its imidazole side chain is pH dependent (pKa ≈ 6), allowing histidine‐rich peptides to switch between active and inactive states depending on the local environment, a property particularly relevant at acidic sites of infection or inflammation [26]. It is important to note that the effects of cationic residue substitutions are sequence‐specific rather than being universally predictable: The number, spacing, and spatial arrangement of charges along the peptide sequence influence membrane selectivity, insertion depth, and the balance between antimicrobial activity and toxicity to eukaryotic cells [27]. Overall, these studies highlight the need to evaluate substitutions of cationic residues within defined peptide scaffolds, rather than inferring effects from net charge alone [28].

This study systematically analyses the impact of substituting Arg, Lys, and His residues in the Lynronne family of AMPs, using synthetic derivatives and in vitro/in silico data to guide their therapeutic optimization against bacteria [29, 30]. Metagenomic screens of gene libraries from the ruminal microbiota led to the identification of three AMPs, named Lynronne‐1, Lynronne‐2, and Lynronne‐3, selected for their broad‐spectrum antibacterial activity associated with low to no toxicity to human cells [31]. These 19–20‐amino‐acid peptides adopt an amphipathic helical conformation in the presence of anionic lipids, with net charges of +6, +5, and +6, respectively, and a hydrophobicity of approximately 40%, characteristics typical of cationic AMPs rich in Arg and Lys. Lynronnes exhibits marked activity against several ESKAPEE pathogens, including methicillin‐resistant *Staphylococcus aureus* (MRSA) and multidrug‐resistant *Pseudomonas aeruginosa* , with MICs often lower for Lynronne‐1 than for Lynronne‐2 and Lynronne‐3 [32]. They rapidly reduce bacterial populations and inhibit biofilm formation, while exhibiting low hemolysis and limited cytotoxicity across various human cell lines [33]. Mechanistic studies demonstrated that the Lynronnes target the bacterial membrane, resulting in rapid bacteriolytic effects. Furthermore, topical administration of Lynronne‐1 (10% w/v) in a murine model of MRSA skin infection reduced bacterial load by 99% [31]. Similarly, Lynronne‐1 (32 mg/kg) and Lynronne‐2 (128 mg/kg) conferred complete protection against *P. aeruginosa* PAO1 infection in the *Galleria mellonella* larvae model [32].

In this context, the Lynronnes derivatives studied here constitute a rational framework for exploring how the nature, arrangement, and proportion of the cationic residues Arg, Lys, and His modulate the activity, toxicity, selectivity, and anti‐biofilm activity of AMPs.

## 2 Materials and Methods

### 2.1 Synthesis, Purification, and Characterization of the Peptides

All peptides were synthesized using standard 9‐fluorenylmethoxycarbonyl solid‐phase peptide synthesis chemistry on Rink amide MBHA resin (0.52 mmol/g). Lynronne‐1, Lynronne‐1R, Lynronne‐1K, Lynronne‐1Inv, Lynronne‐1H, Lynronne‐2, Lynronne‐2R, Lynronne‐2K, Lynronne‐2Inv, and Lynronne‐2H were synthesized manually using HATU (O‐(7‐azabenzotriazol‐1‐yl)‐*N*,*N*,*N*′,*N*′‐tetramethyluronium hexafluorophosphate) as coupling reagent and DIPEA (*N*,*N*‐diisopropylethylamine) as a base. Lynronne‐3, Lynronne‐3K, and Lynronne‐3H were synthesized automatically on a Biotage Initiator+ Alstra peptide synthesizer using HBTU (O‐(benzotriazol‐1‐yl)‐*N*,*N*,*N*′,*N*′‐tetramethyluronium hexafluorophosphate) and DIPEA. Fmoc deprotection was performed with 20% piperidine in DMF (v/v), 2 × 10 min. After chain assembly, cleavage from the resin and sidechain deprotection were carried out using TFA/TIS/H2O (95:2.5:2.5, v/v/v) for 2 h at room temperature. For the Cys‐containing peptides Lynronne‐3, Lynronne‐3K, and Lynronne‐3H, a different cleavage cocktail was used: TFA/thioanisole/EDT/anisole (90:5:3:1, v/v/v/v) to prevent cysteine alkylation. The crude peptides were precipitated in cold diethyl ether, centrifuged, washed with cold diethyl ether, and dried under vacuum. The crude peptides were purified by preparative reversed‐phase HPLC on a Phenomenex Jupiter Proteo, AXIA Packed column (250 × 21.2 mm, 4 μm, 90 Å) using an appropriate gradient of solvents A and B (solvent A: 99.9% water and 0.1% TFA(v/v); solvent B: 90% acetonitrile, 9.9% water, and 0.1% TFA (v/v)) at a flow rate of 10 mL/min. Elution was monitored by UV absorbance at 220 and 280 nm. Peptides were analyzed by analytical reversed‐phase HPLC on a Phenomenex Aeris XB‐C18 column (250 × 4.6 mm) using a linear gradient of increasing acetonitrile concentration (20%–60% B) over 20 min at a flow rate of 1 mL/min. Elution was monitored by UV absorbance at 220 and 280 nm. RP‐HPLC analysis spectrums of the peptides are shown in Figures S1–S13. The molecular masses of the peptides were determined using a SYNAPT G2 HDMS mass spectrometer (Waters) equipped with a pneumatically assisted atmospheric pressure ionization (API) source. The sample was ionized in positive electrospray mode under the following conditions: electrospray voltage, 2.8 kV; orifice voltage, 20 V; nebulizer gas (nitrogen) flow rate, 100 L/h. The mass spectrum (MS) was obtained using a time‐of‐flight (TOF) analyzer. SI‐MS analyses were performed at the Spectropole facility (Aix‐Marseille University, Marseille, France; https://fr‐chimie.univ‐amu.fr/spectropole/). Positive electrospray ionization MSs of the peptides are shown in Figures S1–S13.

**Lynronne‐1** Preparative HPLC: 35%–45% B in 10 min; rt. = 5.2 min. Analytical HPLC: 20%–60% B in 20 min; rt. = 13.1 min; purity: 98.7%. Mass spectrometry (MW: 2399.9 g/mol): mass observed ([M + 6H]^6+^ = 400.9; [M + 5H]^5+^ = 480.9; [M + 4H]^4+^ = 600.9; [M + 3H]^3+^ = 800.8) mass calculated ([M + 6H]^6+^ = 400.1; [M + 5H]^5+^ = 481.0; [M + 4H]^4+^ = 601.0; [M + 3H]^3+^ = 801.0).

**Lynronne‐1R** Preparative HPLC: 30%–60% B in 20 min; rt. = 5.5 min. Analytical HPLC: 20%–60% B in 20 min; rt. = 13.4 min; purity: 95.2%. Mass spectrometry (MW: 2483.9 g/mol): mass observed ([M + 6H]^6+^ = 414.9; [M + 5H]^5+^ = 497.7; [M + 4H]^4+^ = 621.9; [M + 3H]^3+^ = 828.8) mass calculated ([M + 6H]^6+^ = 415.0; [M + 5H]^5+^ = 497.8; [M + 4H]^4+^ = 622.0; [M + 3H]^3+^ = 829.0).

**Lynronne‐1K** Preparative HPLC: 35%–55% B in 20 min; rt. = 6.5 min. Analytical HPLC: 20%–60% B in 20 min; rt. = 12.8 min; purity: 98.9%. Mass spectrometry (MW: 2315.9 g/mol): mass observed (M + 5H]^5+^ = 464.1; [M + 4H]^4+^ = 579.9; [M + 3H]^3+^ = 772.8. [M + 2H]^2+^ = 1158.7) mass calculated ([M + 5H]^5+^ = 464.2; [M + 4H]^4+^ = 580.0; [M + 3H]^3+^ = 773.0; [M + 2H]^2+^ = 1159.0).

**Lynronne‐1Inv** Preparative HPLC: 25%–45% B in 10 min; rt. = 7.5 min. Analytical HPLC: 20%–60% B in 20 min; rt. = 13.0 min; purity: 99.3%. Mass spectrometry (MW: 2399.9 g/mol): mass observed ([M + 5H]^5+^ = 480.9; [M + 4H]^4+^ = 600.9; [M + 3H]^3+^ = 800.8) mass calculated ([M + 5H]^5+^ = 481.0; [M + 4H]^4+^ = 601.0; [M + 3H]^3+^ = 801.0).

**Lynronne‐1H** Preparative HPLC: 33%–43% B in 10 min; rt. = 4.0 min. Analytical HPLC: 20%–60% B in 20 min; rt. = 11.4 min; purity: 99.0%. Mass spectrometry (MW: 2369.7 g/mol): mass observed ([M + 5H]^5+^ = 474.8; [M + 4H]^4+^ = 593.3; [M + 3H]^3+^ = 790.7; [M + 2H]^2+^ = 1185.6) mass calculated ([M + 5H]^5+^ = 475.0; [M + 4H]^4+^ = 593.4; [M + 3H]^3+^ = 790.9; [M + 2H]^2+^ = 1185.8).

**Lynronne‐2** Preparative HPLC: 30%–40% B in 10 min; rt. = 5.4 min. Analytical HPLC: 20%–60% B in 20 min; rt. = 10.7 min; purity: 97.4%. Mass spectrometry (MW: 2433.9 g/mol): mass observed ([M + 5H]^5+^ = 487.7; [M + 4H]^4+^ = 609.4; [M + 3H]^3+^ = 812.2) mass calculated ([M + 5H]^5+^ = 487.8; [M + 4H]^4+^ = 609.5; [M + 3H]^3+^ = 812.3).

**Lynronne‐2R** Preparative HPLC: 25%–40% B in 15 min; rt. = 6.0 min. Analytical HPLC: 20%–60% B in 20 min; rt. = 11.2 min; purity: 98.6%. Mass spectrometry (MW: 2500.0 g/mol): mass observed ([M + 5H]^5+^ = 500.9; [M + 4H]^4+^ = 625.9; [M + 3H]^3+^ = 834.2) mass calculated ([M + 5H]^5+^ = 501.0; [M + 4H]^4+^ = 626.0; [M + 3H]^3+^ = 834.4).

**Lynronne‐2K** Preparative HPLC: 25%–40% B in 15 min; rt. = 7.0 min. Analytical HPLC: 20%–60% B in 20 min; rt. = 10.1 min; purity: 98.7%. Mass spectrometry (MW: 2303.9 g/mol): mass observed ([M + 6H]^6+^ = 384.9; [M + 5H]^5+^ = 461.7; [M + 4H]^4+^ = 576.9; [M + 3H]^3+^ = 768.8; [M + 2H]^2+^ = 1152.8) mass calculated ([M + 6H]^6+^ = 385.0; [M + 5H]^5+^ = 461.8; [M + 4H]^4+^ = 577.0; [M + 3H]^3+^ = 769.0; [M + 2H]^2+^ = 1153.0).

**Lynronne2Inv** Preparative HPLC: 30%–40% B in 10 min; rt. = 4.5 min. Analytical HPLC: 20%–60% B in 20 min; rt. = 10.1 min; purity: 98.8%. Mass spectrometry (MW: 2349.9 g/mol): mass observed ([M + 6H]^6+^ = 392.6; [M + 5H]^5+^ = 470.9; [M + 4H]^4+^ = 588.4; [M + 3H]^3+^ = 784.2; [M + 2H]^2+^ = 1175.7) mass calculated ([M + 6H]^6+^ = 392.7; [M + 5H]^5+^ = 471.0; [M + 4H]^4+^ = 588.5; [M + 3H]^3+^ = 784.3; [M + 2H]^2+^ = 1176.0).

**Lynronne‐2H** Preparative HPLC: 30%–40% B in 10 min; rt. = 3.1 min. Analytical HPLC: 20%–60% B in 20 min; rt. = 9.6 min; purity: 98.3%. Mass spectrometry (MW: 2366.7 g/mol): mass observed ([M + 5H]^5+^ = 474.3; [M + 4H]^4+^ = 592.6; [M + 3H]^3+^ = 789.8; [M + 2H]^2+^ = 1184.1) mass calculated ([M + 5H]^5+^ = 474.3; [M + 4H]^4+^ = 592.7; [M + 3H]^3+^ = 789.9; [M + 2H]^2+^ = 1184.4).

**Lynronne‐3** Preparative HPLC: 30%–40% B in 10 min. rt. = 6.0 min). Analytical HPLC: 20%–60% B in 20 min; rt. = 10.5 min; purity: 96.7%. Mass spectrometry (MW: 2652.1 g/mol): mass observed ([M + 6H]^6+^ = 442.9; [M + 5H]^5+^ = 531.3; [M + 4H]^4+^ = 663.9; [M + 3H]^3+^ = 884.8) mass calculated ([M + 6H]^6+^ = 443.0; [M + 5H]^5+^ = 531.4; [M + 4H]^4+^ = 664.0; [M + 3H]^3+^ = 885.0).

**Lynronne‐3K** Preparative HPLC: 25%–40% B in 15 min; rt. = 6.4 min. Analytical HPLC: 20%–60% B in 20 min; rt. = 9.7 min; purity: 98.6%. Mass spectrometry (MW: 2484.0 g/mol): mass observed ([M + 5H]^5+^ = 497.7; [M + 4H]^4+^ = 621.9; [M + 3H]^3+^ = 828.8) mass calculated ([M + 5H]^5+^ = 497.8; [M + 4H]^4+^ = 622.0; [M + 3H]^3+^ = 829.0).

**Lynronne‐3H** Preparative HPLC: 25%–40% B in 15 min; rt. = 7.1 min). Analytical HPLC: 20%–60% B in 20 min; rt. = 10.0 min; purity: 95.9%. Mass spectrometry: (MW: 2537.8 g/mol): mass observed ([M + 5H]^5+^ = 508.4; [M + 4H]^4+^ = 635.3; [M + 3H]^3+^ = 846.7) mass calculated ([M + 5H]^5+^ = 508.6; [M + 4H]^4+^ = 635.5; [M + 3H]^3+^ = 847.0).

Peptide stock solutions (5 mg/mL) were prepared by dissolving the lyophilized peptides in sterile water. Peptide concentrations were determined from UV absorption spectra recorded over 250–340 nm using the multiwavelength fitting method described by Siemann et al. [28]. Briefly, the experimental spectra were fitted to simulated spectra of native and unfolded protein states to determine peptide concentrations. Although the peptides contain a tryptophan (Trp) chromophore and can therefore be quantified from their absorbance at 280 nm, the multiwavelength approach was used to exploit the information across the measured spectral range rather than relying on a single wavelength. This approach applies the Beer–Lambert law and estimates concentration from the spectral contributions of the absorbing groups present in each peptide sequence. Stock solutions were aliquoted and stored at −80°C until use.

### 2.2 Circular Dichroism (CD) and Secondary Structure Determination

Peptide samples for CD were prepared as ~2.5 mg/mL stock solutions by dissolving peptide lyophilized powder in sterile water. The precise concentration of each peptide solution was quantified as described above. CD samples contained ~65 μg/mL peptide concentration in 10 mM sodium phosphate buffer at pH 7.4, in the absence or in the presence of 20 mM SDS (above the critical micelle concentration). CD spectra were recorded on a Jasco J‐815 spectropolarimeter. Measurements were carried out at 25°C using a quartz cell with a 1‐mm path length. Spectra were recorded from 185 to 270 nm with a bandwidth of 1 nm, a step size of 0.2 nm, a scanning speed of 20 nm/min, and were averaged over four scans. Control spectra without peptide were recorded and subtracted from peptide spectra. To calculate the percentages of secondary structures, CD spectra were deconvoluted using ChiraKit and SELCON3 or SELCON2 analysis methods [34]. For secondary structure quantification, the raw CD spectra (baseline‐corrected) were deconvoluted using ChiraKit with the SELCON3 or SELCON2 analysis method. No smoothing was applied prior to deconvolution. For visualization purposes only, representative CD spectra shown in figures were smoothed using a 3‐point smoothing function as implemented in ChiraKit (https://spc.embl‐hamburg.de/app/chirakit). This smoothing did not affect the reported secondary structure percentages, which were derived exclusively from the unsmoothed data.

### 2.3 Bacterial Strains and Growth Conditions

The following ESKAPEE reference strains were used: *Acinetobacter baumannii* (ATCC 19606), *Enterobacter cloacae* (ATCC 13047), *Escherichia coli* (ATCC 8739), *Enterococcus faecalis* (ATCC 29212), *Enterococcus faecium* (ATCC 19434), *Klebsiella pneumoniae* (ATCC 700603), *Pseudomonas aeruginosa* (ATCC 9027), and *Staphylococcus aureus* (ATCC 6538). Pathogenic and clinical *E. coli* strains comprised enteropathogenic *E. coli* (EPEC) and enterohemorrhagic *E. coli* (EHEC), both provided by Prof Brendan Kenny (Bristol University, United Kingdom) [35]; the adherent‐invasive *E. coli* reference strain LF82 (provided by Eric Oswald (IRSD, Toulouse, France); and the extended spectrum β‐lactamase (ESBL) producer, amoxicillin‐resistant uropathogenic isolate (UPEC) CF1702 (DSM 22314, DSMZ, Braunschweig, Germany). These strains were handled under biosafety level 2 (BSL‐2) containment. Unless otherwise stated, strains were routinely cultured in Mueller–Hinton (MH) agar at 37°C under aerobic conditions. *E. faecalis* and *E. faecium* were incubated under microaerobic conditions using the BD GasPak EZ system (Becton Dickinson, Le Pont‐de‐Claix, France). A panel of Risk Group 3 biothreat agents was also included: *Bacillus anthracis* Ames, *Burkholderia pseudomallei* K96243, *Burkholderia mallei* ATCC 23344, *Brucella melitensis* (NR‐256), *Francisella tularensis* (NR‐643), and *Yersinia pestis* (NR‐641). These strains were handled under biosafety level 3 (BSL‐3) containment at the IRBA (Brétigny‐sur‐Orge, France). Risk Group 3 agents were cultured on Chocolate agar PolyViteX (Biomerieux) at 37°C, except *Y. pestis* , which was incubated at 26°C. The mycobacterial pathogenic strains, *Mycobacterium abscessus* CIP104536T smooth (S) or rough (R) morphotype, *Mycobacterium marinum* ATCC BAA‐535/M, and the avirulent *M. tuberculosis* mc^2^6230 reference strains were routinely grown in Middlebrook 7H9 broth (BD Difco, Le Pont de Claix, France) supplemented with 0.2% glycerol (Euromedex, Souffelweyersheim, France); 0.05% Tween‐80 (Sigma‐Aldrich, Saint‐Quentin Fallavier, France); 10% oleic acid, albumin, dextrose, catalase (OADC enrichment; BD Difco) (7H9‐S^OADC^); and 24 μg/mL d‐panthothenate (for *M. tuberculosis* mc^2^6230). All cultures were maintained at 37°C with gentle shaking, except for *M. marinum* , which was cultured at 32°C.

### 2.4 Minimum Inhibitory Concentration (MIC) Determination

MICs against the BSL‐2 ESKAPEE panel were determined by broth microdilution adapted from the EUCAST reference procedure, as previously described [36]. Single colonies were inoculated into 3 mL of MH broth and incubated for approximately 16 h at 37°C with shaking (200 rpm). Overnight cultures were diluted 1:100 in fresh MH and grown to mid‐exponential phase (OD_600nm_ ≈ 0.6), then adjusted to 5 × 10^5^ CFU/mL in MH. Aliquots of 100 μL of bacterial suspension dispensed into sterile polypropylene 96‐well microplates (Greiner BioOne, Dominique Dutscher, Brumath, France). Bacteria were exposed to increasing concentrations of peptides obtained by two‐fold serial dilutions. Plates were incubated at 37°C for 18–24 h, and the MIC was defined as the lowest peptide concentration that produced no visible growth. All strains were tested under aerobic conditions, except *E. faecalis* and *E. faecium*, which were incubated in a microaerobic atmosphere generated using the BD GasPak system (Sigma‐Aldrich, Lyon, France). Amoxicillin and tetracycline were tested in parallel as reference antimicrobials (full details of MIC values of peptides and antibiotics are given in the Supporting Information, in Tables S1 to S6). For the BSL‐3 bacterial strains, MICs were determined following the Clinical and Laboratory Standards Institute (CLSI) recommendations, as previously described [37]. Lynronnes were tested from 250 to 0.24 μg/mL using twofold serial dilutions. Ciprofloxacin was included as a reference antimicrobial in each experiment. To evaluate salt tolerance and effect of human serum, MIC assays were repeated in MH broth supplemented with physiological concentrations of NaCl (150 mM), MgCl_2_ (1 mM), CaCl_2_ (2 mM), or a combination of all three salts (150 mM NaCl, 1 mM MgCl_2_, 2 mM CaCl_2_) or supplemented with 50% human serum (obtained from Sigma Aldrich). Standard MH broth (without added salt) served as the control. Regarding mycobacterial strains, the antimycobacterial activity of the different peptides was determined using the microdilution method in sterile 96‐well flat‐bottom Greiner Bio‐One polypropylene microplates with lid (Thermo Fisher Scientific) using the resazurin microtiter assay (REMA) as previously described [38, 39]. Briefly, log‐phase bacteria were diluted to 5 × 10^6^ CFU/mL in appropriate 7H9‐S^OADC^ medium. Then, 100 μL of this inoculum was added to each well containing 100 μL of 7H9‐S^OADC^, serial two‐fold dilutions of the peptides or controls, to a final volume of 200 μL. Growth controls containing no inhibitor (i.e., bacteria only), inhibition controls containing 50 μg/mL kanamycin (Euromedex; #UK0010D), and sterility controls (i.e., medium only) without inoculation were also included. Microplates were incubated at 37°C (32°C for *M. marinum* ) in a humidity chamber to prevent evaporation for 3–5 days ( *M. abscessus* ) or 10–14 days ( *M. marinum* and *M. tuberculosis* ). Then, 20 μL of a 0.02% (w/v) resazurin solution in sterile water (Sigma‐Aldrich; #R7017) was added to each well, and the plates were incubated at 37°C (or 32°C) until color change from blue to pink or violet in the control well (i.e., bacteria alone). Relative fluorescence units (RFU) of the metabolite resorufin (λex/λem = 530/590 nm) were quantified using a Tecan Spark 10 M multimode microplate reader (Tecan Group Ltd., France). RFU were defined as: RFU% = (test well FU/mean FU of control B wells) × 100. MIC values were determined by fitting the RFU% sigmoidal dose–response curves in the Kaleidagraph 4.2 software. The lowest compound concentration leading to 90% inhibition of bacterial growth was defined as the MIC. Amikacin and isoniazid were used as control antibiotics for mycobacteria. All MIC measurements were performed independently three times (*n* = 3).

### 2.5 Cytotoxic Assay on Human Cells

The toxicity of the peptides was evaluated in human cells as previously described [40]. Human cells tested correspond to lung (BEAS‐2B cells from ECACC, Sigma Aldrich, France), intestine (Caco‐2 cells (ATCC HTB‐37) from ATCC, Europe), skin (HaCaT cells from Creative Bioarray, Shirley, NY 11967, United States), and liver (HepG2 cells (ATCC HB‐8065 from ATCC, Europe). Cells were routinely maintained in Dulbecco's Modified Essential Medium (DMEM) supplemented with 10% fetal bovine serum (FBS), 1% L‐glutamine, and 1% antibiotics (penicillin and streptomycin) (all from Thermo Fisher Scientific, Illkirch‐Graffenstaden, France). Cells were grown on 75 cm^2^ flasks in a 5% CO_2_ incubator at 37°C. Before assessing toxicity, cells were detached from cell culture containers with trypsin–EDTA solution (Thermo Fisher Scientific, France). Cell density was measured using a Malassez counting chamber before cells were seeded into 96‐well cell culture plates (Greiner bio‐one, France) at approximately 10,000 cells per well. Once confluency was reached (2 days), the media in the wells were replaced, and cells were treated with increasing concentrations of peptides diluted in culture media. After 48 h incubation at 37°C in a 5% CO_2_ incubator, cell viability was measured using resazurin. Wells were emptied, and cells were treated with 100 μL of resazurin diluted to 30 μg/mL in sterile PBS containing calcium and magnesium (PBS^++^, pH 7.4). After 2 h incubation at 37°C, fluorescence intensity (excitation wavelength of 530 nm/emission wavelength of 590 nm) was measured using a microplate reader (Biotek, Synergy Mx, France). The fluorescence values were normalized by the negative control corresponding to untreated cells receiving vehicle alone (sterile distilled water). Triton X‐100 at 0.1% (v/v) was used as a positive control for cytotoxicity, yielding 100% toxicity. The cytotoxic concentration 50 (i.e., CC_50_) values of peptides corresponding to the concentrations that caused a reduction of 50% in cell viability were calculated using the GraphPad Prism 11 software. Experiments were performed in independent triplicate (*n* = 3).

### 2.6 Hemolytic Activity Assay

Hemolytic activity of peptides was evaluated using commercial human red blood cells (hRBCs) (from Divbio Science Europe, NL) as previously described [41]. hRBCs were washed with sterile phosphate‐buffered saline (PBS, pH 7.4) and collected by centrifugation at 800 × g for 5 min. Washed hRBCs were resuspended at 8% (v:v) in DMEM without phenol red supplemented with 10% FBS. The hRBC suspension (100 μL) was then added to sterile 96‐well microplates already containing 100 μL of peptides at increasing concentrations, diluted in culture medium. After 1 h at 37°C, the plates containing hRBC were centrifuged at 800 g for 5 min, and 100 μL of supernatant were carefully transferred to new 96‐well microplates. The absorbance was measured at 415 nm using a microplate reader (SynerMix, Biotek). Triton X‐100 at 0.1% (v/v) was used as a positive control giving 100% hemolysis. The hemolytic concentration 50 (i.e., HC_50_) values of compounds corresponding to the concentrations that caused 50% of hemolysis were calculated using the GraphPad Prism 11 software. Experiments were performed in independent triplicate (*n* = 3).

### 2.7 Membrane Permeabilization Assay

Membrane permeabilization was assessed by measuring ATP release using a bioluminescence assay based on the luciferin/luciferase reaction as previously described [42]. *Escherichia coli* and *S. aureus* were used as models and were grown to mid‐exponential phase (OD₆₀₀ = 0.5–0.6) in MH broth at 37°C with agitation (200 rpm). The bacterial final inoculum was adjusted to approximately 10^7^ CFU/mL. For each assay, 50 μL of bacterial suspension was mixed with 50 μL of solution containing the peptide (at 4× MIC) and the ATP measurement reagent following instructions from the supplier (commercial ATP assay kit from Yelen, France) [42]. Assay was performed in a white opaque 96‐well plates to minimize background luminescence. ATP release was expressed as a percentage of the kit's positive control, which causes complete membrane lysis and yields 100% ATP release. Untreated bacteria served as the negative control. All assays were performed in independent triplicate (*n* = 3).

### 2.8 Antibiofilm Activities

Overnight cultures of *E. coli* LF82 or *S. aureus* (ATCC 6538) in MH broth were incubated until mid‐exponential phase at 37°C, 200 rpm. For biofilm inhibition assay, cultures were diluted to OD600nm = 0.1 in fresh MH broth, and 50 μL were aliquoted into 96‐well polystyrene flat‐bottom plates (Greiner BioOne, Dominique Dutscher, Brumath, France). PAM solution (50 μL) at twice the indicated final concentration, or MH broth alone as a no‐peptide control, was added simultaneously, resulting in a final OD600nm of 0.05 in a total volume of 100 μL. Plates were statically incubated at 37°C for 24 h. For biofilm destruction assay, cultures of *E. coli* LF82 or *S. aureus* ATCC 6538 were diluted to OD_600nm_ = 0.05 in fresh MH broth, and 100 μL was aliquoted into 96‐well polystyrene flat‐bottom (Greiner BioOne, Dominique Dutscher, Brumath, France). Plates were statically incubated at 37°C for 24 h to allow biofilm formation. Planktonic cells were removed by inversion and washed by three successive baths in deionized water. Fresh MH broth (100 μL) supplemented with PAM at the indicated concentrations, or MH broth alone as a no‐peptide control, was added, and plates were incubated for an additional 24 h at 37°C. In both assays, at the end of the incubation, planktonic cells were removed by inversion and surface‐attached cells were washed by three successive baths in deionized water. Plates were then incubated at 65°C for 1 h to allow biofilm to dry and be fixed. Each well was then supplemented with 100 μL of 1% crystal violet solution and incubated for 15 min. The crystal violet solution was removed by inversion, and the wells were washed with three successive baths in deionized water. Then, 120 μL of 30% acetic acid was added to dissolve the stained biofilms, and absorbance at 570 nm was measured using a microplate reader (Synergy H1, BioTek). Experiments were performed in independent triplicate (*n* = 3).

### 2.9 Data Visualization and Statistical Analysis

Data visualization and statistical analyses were performed using GraphPad Prism version 11.1.0 for Windows (GraphPad Software, Boston, MA, United States; https://www.graphpad.com). Statistical tests are specified in the corresponding figure legends. Selected figures (Figures 3, 4, 5, 6 and 9, 10, 11) were generated in Python [43, 44, 45] version 3.12.14 using Matplotlib version 3.10.8. Data organization and numerical processing were performed using pandas version 2.2.3 and NumPy version 2.3.5.

## 3 Results

### 3.1 Peptide Design and Synthesis

For each Lynronne (1–3), derivatives were designed by replacing the native cationic residues (Arg or Lys or His) with (i) all‐Arg, (ii) all‐Lys, (iii) reverse Arg‐Lys (“Inv” derivatives), and (iv) all‐His. For Lynronne‐3, whose native sequence contains only Arg, only two derivatives were generated (all‐Lys and all‐His) (Table 1).

**TABLE 1:** Sequences of the peptides used in this study. Cationic residues are shown in red, and substituted residues are underlined.

Peptides	Sequence
Lynronne‐1	LPRRNRWSKIWKKVVTVFS‐NH_2_
Lynronne‐1R	LPRRNRWS R IW RR VVTVFS‐NH_2_
Lynronne‐1K	LP KK N K WSKIWKKVVTVFS‐NH_2_
Lynronne‐1Inv	LP KK N K WS R IW RR VVTVFS‐NH_2_
Lynronne‐1H	LP HH N H WS H IW HH VVTVFS‐NH_2_
Lynronne‐2	HLRRINKLLTRIGLYRHAFG‐NH_2_
Lynronne‐2R	R LRRIN R LLTRIGLYRR AFG‐NH_2_
Lynronne‐2K	K L KK INKLLT K IGLY KK AFG‐NH_2_
Lynronne‐2Inv	HL KK IN R LLT K IGLY KHAFG‐NH_2_
Lynronne‐2H	H L HH IN H LLT H IGLY HH AFG‐NH_2_
Lynronne‐3	NRFTARFRRTPWRLCLQFRQ‐NH_2_
Lynronne‐3K	N K FTA K F KK TPW K LCLQF K Q‐NH_2_
Lynronne‐3H	N H FTA H F HH TPW H LCLQF H Q‐NH_2_

A total of 13 peptides, parental peptides and their derivatives shown in Table 1, were synthesized by standard Fmoc solid‐phase peptide synthesis. The identity of each peptide was confirmed by ESI‐MS, and analytical RP‐HPLC showed purities above 95% for all final products. Detailed synthetic procedures, purification methods, and analytical characterization are provided in the Experimental Section and in Figures S1–S13.

Helical wheel projections of the parental Lynronne‐1, Lynronne‐2, and Lynronne‐3 sequences, with the positions modified in the corresponding analogues highlighted, are shown in Figure 1 to illustrate the spatial distribution of hydrophobic and polar residues under an ideal α‐helical conformation.

**FIGURE 1:**
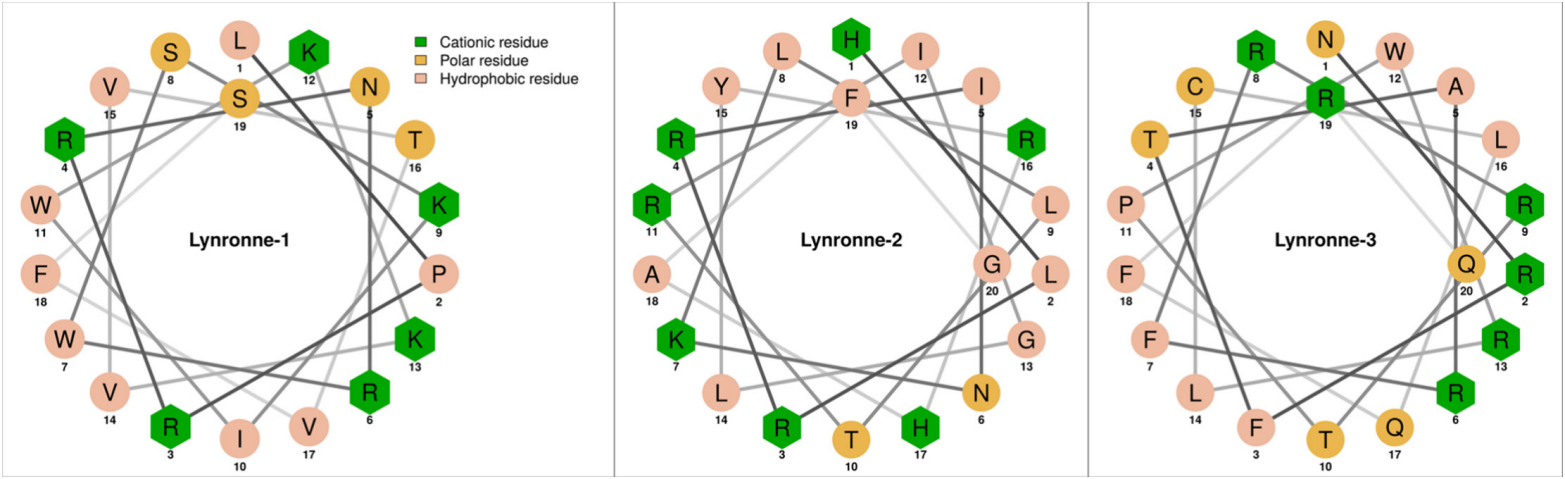
Helical wheel projections of the parental Lynronne peptides. Lynronne‐1, Lynronne‐2, and Lynronne‐3. Projections assume an ideal α‐helical conformation. Hexagonal green cationic residues indicate positions modified in the corresponding peptide derivatives. Helical wheel projections were generated using NetWheels: Peptides Helical Wheel and Net projections maker.

Throughout this study, peptide names refer exclusively to members of the Lynronne family and should not be confused with unrelated AMPs bearing similar designations. For clarity, abbreviations used in the figures, L1, L2, and L3 correspond to Lynronne‐1, Lynronne‐2, and Lynronne‐3, respectively, with derivative suffixes retained where applicable.

### 3.2 Structural Study

Lynronne‐1, Lynronne‐2, and Lynronne‐3 were reported to adopt amphipathic α‐helical conformations in membrane‐mimetic environments [46, 47]. Accordingly, PEP‐FOLD [48] modelling predicts α‐helices for all three peptides, whereas NMR spectroscopy of Lynronne‐1 confirms a 13‐residue amphipathic helix encompassing all six cationic residues [32, 47].

The conformational propensities of the Lynronnes derivatives were investigated by far‐UV CD (185–270 nm) in phosphate buffer (pH 7.4), in the absence and presence of SDS micelles (at 20 mM) as a membrane‐mimicking environment. This technique measures the differential absorption of right‐ and left‐circularly polarized light, enabling the identification of secondary‐structure elements such as α‐helices, β‐sheets, and disordered conformations. α‐Helical structures are characterized by diagnostic negative bands at 208 and 222 nm, whereas antiparallel β‐sheet conformations display a negative band around 218 nm and a positive band near 195 nm. In contrast, disordered structures exhibit low ellipticity above 210 nm and a negative band near 195–200 nm [49, 50]. The secondary structure of the peptides was estimated by deconvolution of their CD spectra using the open‐access online tool Chirakit [51]. In phosphate buffer, the CD spectra indicated that the Lynronnes derivatives are predominantly unstructured, as evidenced by a negative minimum around 200 nm. Spectral deconvolution confirmed that disordered conformations were dominant, with minor contributions from other structural elements, such as β‐strands, turns, and, to a lesser extent, α‐helical segments. In contrast, in the presence of anionic SDS micelles, and in agreement with previous predictions and NMR data reported for Lynronne‐1, a marked increase in α‐helical content was observed, as indicated by the characteristic double minima at approximately 208 and 222 nm (Figure 2).

**FIGURE 2:**
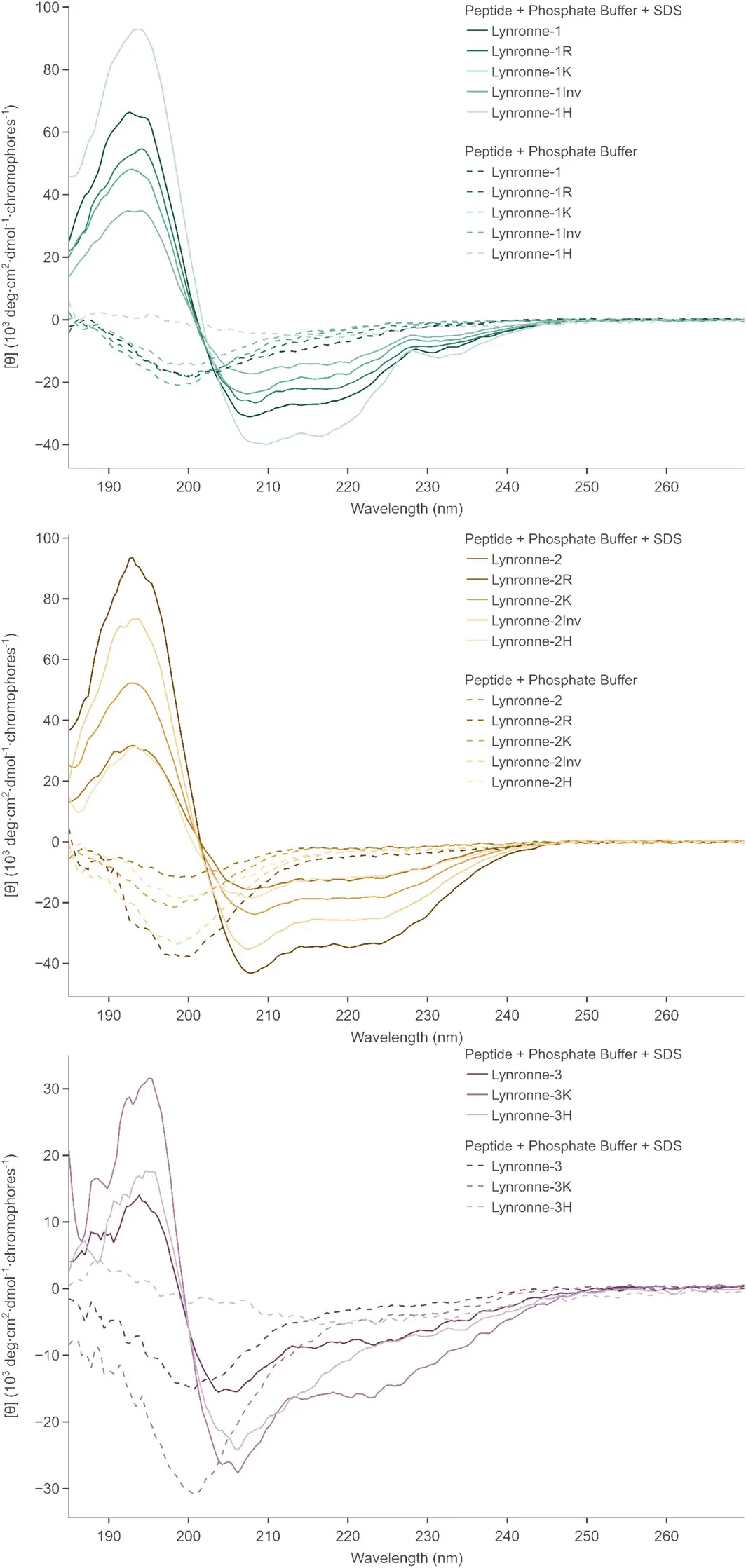
Circular dichroism (CD) spectra of the Lynronnes and their derivatives. CD spectra of the peptides (at 65 μg/mL) were recorded in 20 mM sodium phosphate buffer (pH 7.4) in the absence or presence of 20 mM SDS. Each CD spectrum was recorded between 185 and 270 nm and represents the average of four scans.

Among the parental peptides, Lynronne‐1 displayed the highest helical propensity in the presence of SDS (80.2%), closely followed by Lynronne‐2 (77.0%), whereas Lynronne‐3 showed significantly lower helical content (33.7%) (Table 2). Substituting the cationic residues with Arg, Lys, or His altered the α‐helix percentage but preserved the overall helical fold. For example, the reversed derivative Lynronne‐1Inv, in which only the positions of Arg and Lys are inverted, exhibited 47.4% helicity compared with 80.2% for parental Lynronne‐1, indicating that even subtle changes in the arrangement of cationic residues can markedly affect secondary structure. Similarly, all‐Arg variant Lynronne‐2R showed 43.2% helicity compared with 77.0% for parental Lynronne‐2. In the Lynronne‐3 scaffold, all‐Lys variant Lynronne‐3K displayed 46.0% helicity compared with 33.7% for parental Lynronne‐3. However, no consistent trend emerged across the three Lynronnes scaffolds, indicating that the effect of each substitution depends on the specific sequence context (Table 2).

**TABLE 2:** Secondary structure content (%) of the parental Lynronnes and their derivatives. Percentages of secondary structure contents of peptides in sodium phosphate buffer (pH 7.4), with and without SDS micelles, were determined from their respective CD spectra shown in Figure 2.

Sample	Alpha helix	Beta sheet	Turns	Disordered
Lynronne‐1 Buffer	15.7	21.6	15.3	47.4
Lynronne‐1 Buffer + SDS	80.2	0.0	2.6	17.2
Lynronne‐1R Buffer	11.0	24.0	15.8	49.2
Lynronne‐1R Buffer + SDS	72.6	0.0	7.0	20.4
Lynronne‐1K Buffer	9.4	23.1	15.6	51.9
Lynronne‐1K Buffer + SDS	63.9	3.0	9.5	23.6
Lynronne‐1Inv Buffer	10.1	24.5	15.9	49.5
Lynronne‐1Inv Buffer + SDS	47.4	13.0	11.0	28.6
Lynronne‐1H Buffer	15.9	36.6	9.6	37.9
Lynronne‐1H Buffer + SDS	77.4	0.0	7.2	15.4
Lynronne‐2 Buffer	13.1	8.4	20.1	58.4
Lynronne‐2 Buffer + SDS	77.0	0.0	7.6	15.4
Lynronne‐2R Buffer	12.1	25.4	16.8	45.7
Lynronne‐2R Buffer + SDS	43.2	15.5	10.7	30.6
Lynronne‐2K Buffer	9.9	21.1	17.7	51.3
Lynronne‐2K Buffer + SDS	68.4	0.5	7.7	23.4
Lynronne‐2Inv Buffer	8.9	16.2	18.7	56.2
Lynronne‐2Inv Buffer + SDS	79.3	0.0	6.4	14.3
Lynronne‐2H Buffer	13.9	18.7	18.3	49.1
Lynronne‐2H Buffer + SDS	45.6	10.0	13.1	31.3
Lynronne‐3 Buffer	12.0	23.3	15.9	48.8
Lynronne‐3 Buffer + SDS	33.7	12.0	14.9	39.4
Lynronne‐3K Buffer	12.4	17.0	19.5	51.1
Lynronne‐3K Buffer + SDS	46.0	0.0	25.7	28.3
Lynronne‐3H Buffer	5.5	26.1	17.3	51.1
Lynronne‐3H Buffer + SDS	39.3	7.5	16.7	36.5

### 3.3 Antimicrobial Activity

The antimicrobial activity of all peptides was first evaluated the ESKAPEE panel (Figure 3A). MIC values were determined by broth microdilution over a concentration range of 1–500 μg/mL. The all‐histidine variants of each Lynronne were inactive against all tested strains at concentrations up to 500 μg/mL. In contrast, the other derivatives displayed variable activities depending on both the bacterial strain and the Lynronnes scaffold. *Escherichia coli* was the most sensitive strain, with an 8‐fold lower MIC for Lynronne‐2K (0.98 μg/mL) than for parental Lynronne‐2 (7.81 μg/mL). Against Gram‐positive bacteria, the Lynronne‐2R variant showed improved activity relative to Lynronne‐2. However, the effect of substituting cationic residues was strongly strain‐dependent and sequence‐specific, and no consistent trend could be established across all scaffolds from these initial results (Figure 4A).

**FIGURE 3:**
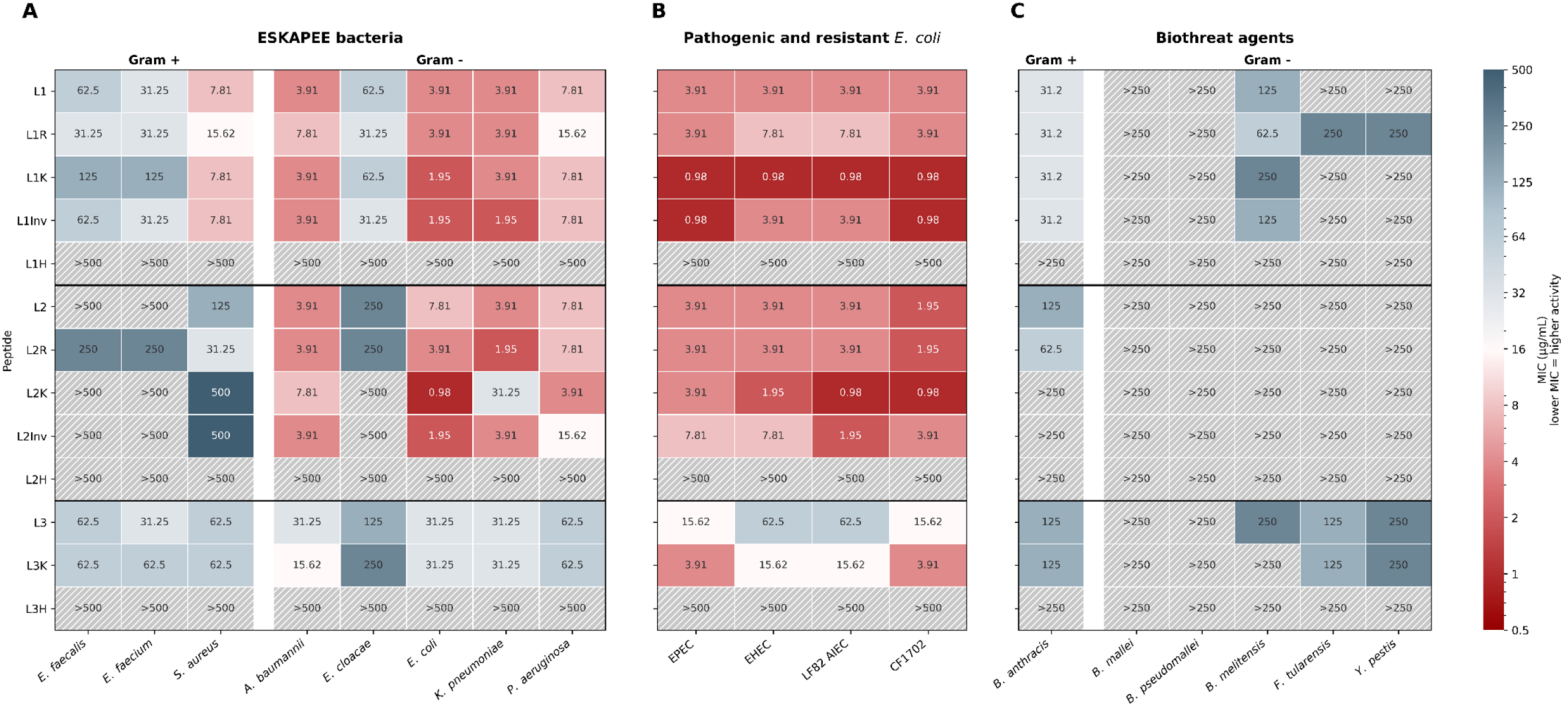
Antibacterial activity landscape of the parental Lynronnes and their derivatives. In this figure, L1, L2, and L3 denote Lynronne‐1, Lynronne‐2, and Lynronne‐3, respectively; derivative labels follow the same convention (e.g., L1R denotes Lynronne‐1R). MIC values are shown in μg/mL. Dark red indicates greater antibacterial activity, corresponding to a lower MIC, whereas blue indicates lower activity with higher MIC. Hatched grey cells indicate that the MIC exceeded the highest concentration tested (i.e., > 500 μg/mL). Horizontal divisions separate the L1, L2 and L3 families. MIC determination was performed in independent triplicates (*n* = 3).

**FIGURE 4:**
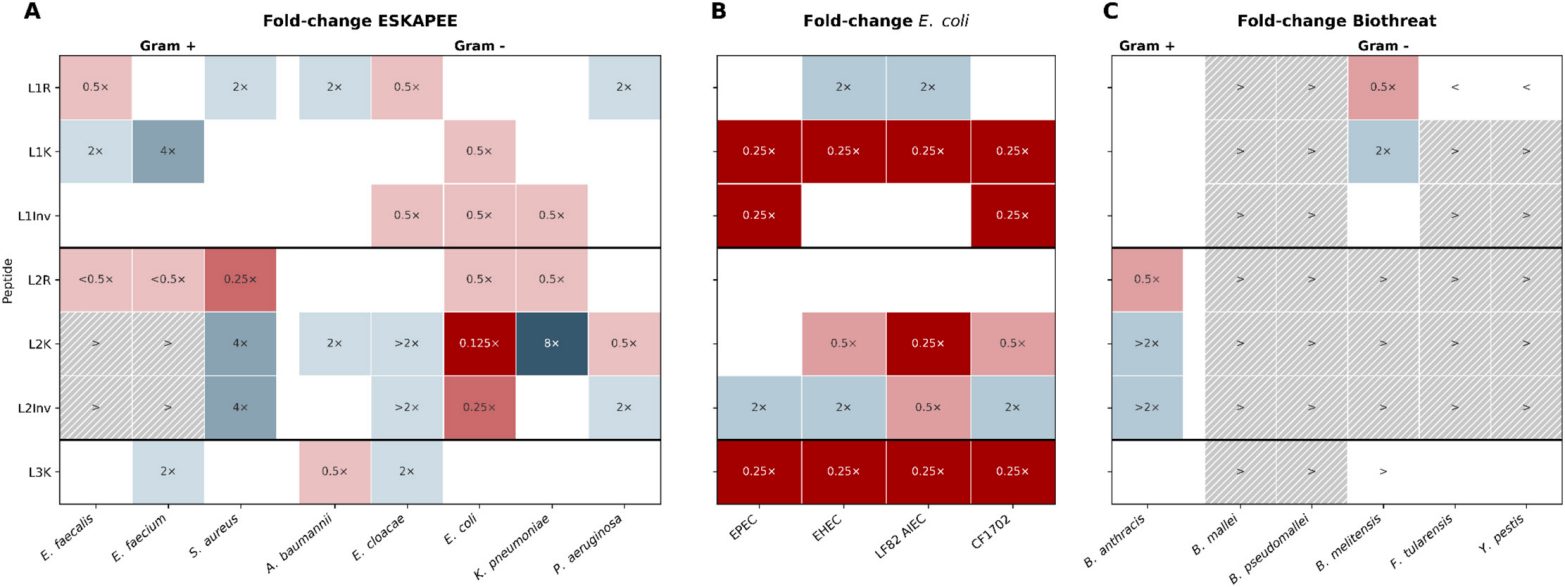
Fold changes in MIC of each derivative compared with the corresponding parental peptides. In this figure, L1, L2, and L3 denote Lynronne‐1, Lynronne‐2, and Lynronne‐3, respectively; derivative labels follow the same convention (e.g., L1R denotes Lynronne‐1R). Fold changes in MIC of each derivative were calculated by dividing their MIC values by the ones of the corresponding parental peptide. Red color indicates increased activity compared with parental peptide, blue color indicates decreased activity compared with the parental peptide, white color indicates an MIC equal to that of the corresponding parental peptide, and hatched grey cells indicate that the MIC exceeded the highest concentration tested (i.e., > 500 μg/mL). MIC determination was performed in independent triplicate (*n* = 3).

Given the potent activity observed against *E. coli* reference strain and the clinical importance of this species, further testing was performed on four pathogenic or resistant isolates of *E. coli* , that is, the enteropathogenic *E. coli* (EPEC), the enterohemorrhagic *E. coli* (EHEC), the adherent‐invasive *E. coli* reference strain LF82, and the ESBL producer amoxicillin‐resistant uropathogenic isolate (UPEC) CF1702 (Figure 3B). All peptides except the all‐His variants showed good activity, with MICs ranging from 0.98 to 62.50 μg/mL. Notably, the Lynronne‐1K variant reached an MIC of 0.98 μg/mL against all four strains, exceeding the activity observed against the ATCC 8739 *E. coli* strain. Across the three Lynronnes scaffolds, the all‐Lys variants consistently showed improved activity relative to their parental counterparts, suggesting that lysine residues may be particularly favorable for anti‐ *E. coli* activity (Figure 4B).

As part of the evaluation of their antibacterial spectrum, the Lynronnes derivatives were further assessed against a panel of priority biothreat agents, including the Gram‐positive *Bacillus anthracis* and the Gram‐negative species *Burkholderia mallei* , *Burkholderia pseudomallei* , *Brucella melitensis* , *Francisella tularensis* , and *Yersinia pestis* (Figure 3C). Overall, the peptides exhibited species‐dependent antibacterial activity, with MICs ranging from 31.25 to > 250 μg/mL (Figure 3C). The greatest and most consistent activity was observed against *B. anthracis* , particularly among the Lynronne‐1‐derived peptides, all of which reached an MIC value of 31.25 μg/mL except the all‐His variant. In contrast, none of the peptides inhibited the growth of *B. mallei* or *B. pseudomallei* even at the highest concentration tested. Among the Gram‐negative species tested, antibacterial activity was limited to a subset of peptides, with Lynronne‐1R showing enhanced activity against *B. melitensis* , *F. tularensis* , and *Y. pestis* relative to the parental Lynronne‐1 peptide (Figure 4C). The all‐His variants were inactive against all biothreat agents tested, indicating that histidine substitution was detrimental to antibacterial activity in this context.

The antimicrobial activity of the Lynronnes parent molecules and their cationic amino acid variants was finally evaluated against *Mycobacterium abscessus* ( *M. abscessus* S and R), *Mycobacterium tuberculosis* mc^2^6230 ( *M. tuberculosis* ), and *Mycobacterium marinum* ( *M. marinum* ) (Figure 5). Lynronne‐1 exhibited the broadest, albeit moderate, activity, with the lowest MIC against *M. tuberculosis* (291 ± 19 μg/mL). Substitution of cationic amino acids with arginine in Lynronne‐1R improved its potency against *M. tuberculosis* by 1.6‐fold (179 ± 3 μg/mL), whereas lysine substitutions (e.g., Lynronne‐1K) retained moderate activity. Notably, Lynronne‐1 and Lynronne‐1K were the only peptides active against *M. abscessus* and *M. marinum*, with Lynronne‐1K showing MIC values of 717 ± 20 μg/mL and 552 ± 52 μg/mL, respectively. In contrast, all‐His and reverse derivatives (Inv) showed abolished or reduced activity across all strains. Overall, all‐Arg derivatives emerged as the most effective modification for improving antimicrobial potency, particularly against *M. tuberculosis* .

**FIGURE 5:**
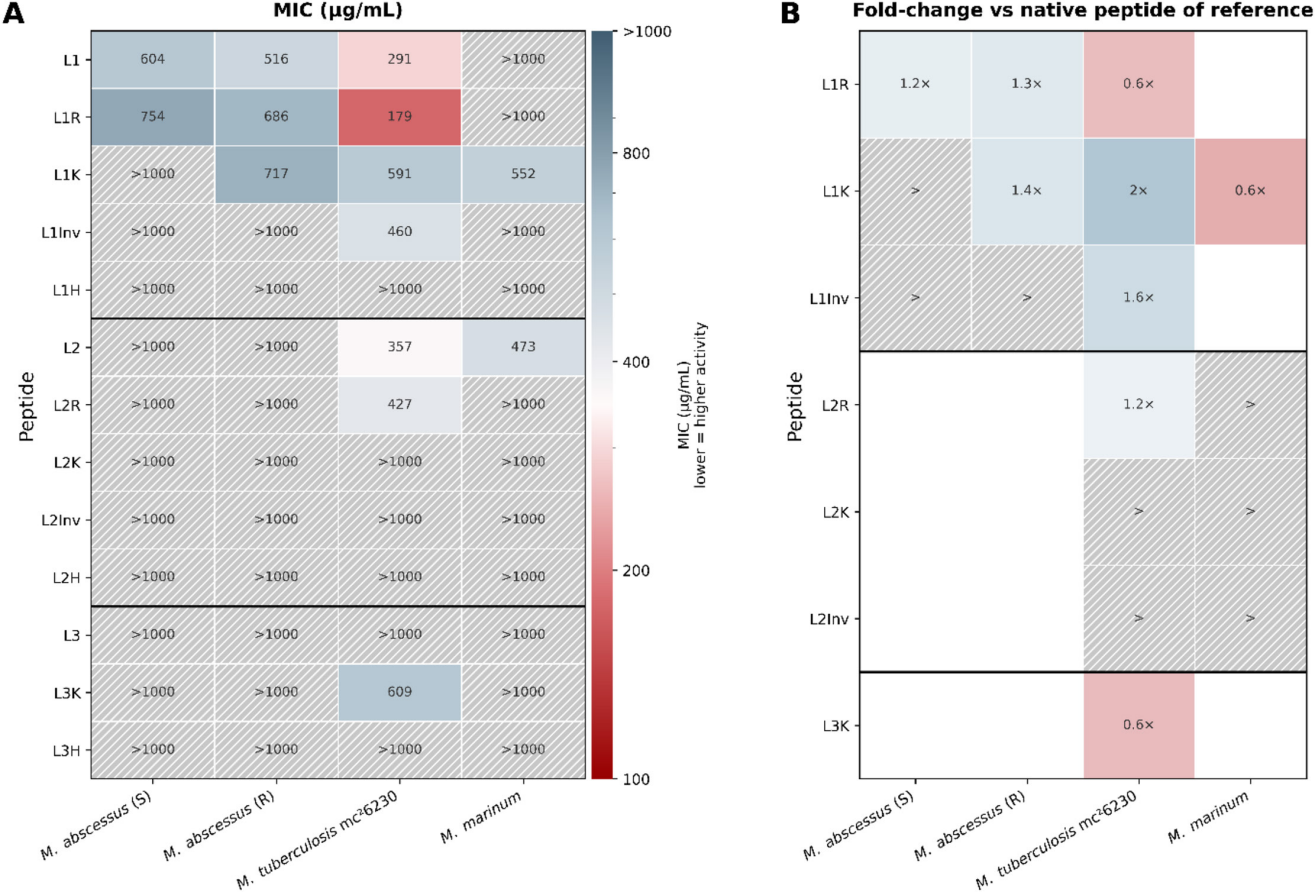
Antibacterial activity landscape and fold changes in MIC of each derivative compared with the corresponding parental peptides against mycobacterial strains. In this figure, L1, L2, and L3 denote Lynronne‐1, Lynronne‐2, and Lynronne‐3, respectively; derivative labels follow the same convention (e.g., L1R denotes Lynronne‐1R). (A) MIC values are shown in μg/mL. Dark red indicates greater antibacterial activity, corresponding to a lower MIC, whereas blue indicates lower activity. White color indicates an MIC equal to that of the corresponding parental peptide. Hatched grey cells indicate that the MIC exceeded the highest concentration tested (i.e., > 1000 μg/mL). Horizontal divisions separate the L1, L2, and L3 families. (B) Fold changes in MIC of each derivative were calculated by dividing their MIC values by the ones of the corresponding parental peptide. MIC determination was performed in independent triplicates (*n* = 3).

Because salt sensitivity is a major limitation of many AMPs, the antibacterial activity of the Lynronnes derivatives was evaluated in the presence of physiological concentrations of NaCl (150 mM), CaCl_2_ (2 mM), and MgCl_2_ (1 mM). From this point, based on their highest sensitivity to the peptides, we focused on one Gram‐negative ( *E. coli* ATCC 8739) and one Gram‐positive ( *S. aureus* ATCC 6538) strain as model organisms to further characterize activity and mechanisms of action. Parental and derivatives peptides were tested, with the exception of all‐His derivatives that are not active. The MICs determined under these conditions are summarized in Figure 6A,B. Overall, most derivatives showed reduced antibacterial activity under high‐salt conditions. The all‐Lys variants were the most salt‐sensitive, with MIC increasing by 16‐ to 500‐fold depending on the peptide scaffold and bacterial strain. For example, the MIC of Lynronne‐2K against *E. coli* increased 500‐fold (from 0.98 to 500 μg/mL), in the presence of each salt, whereas the parental Lynronne‐2 exhibited a 32‐fold increase (from 7.81 to 250 μg/mL). In contrast, all‐Arg derivatives were generally less affected by salt supplementation. Notably, Lynronne‐1R exhibited only an eightfold increase in MIC against *E. coli* and even showed better activity against *S. aureus* in the presence of salts (from 15.62 to 7.81 μg/mL). An exception to this trend was observed for the Lynronne‐3 scaffold, where the all‐Lys variant (Lynronne‐3K) retained greater activity than its all‐Arg counterpart against *S. aureus* in the presence of MgCl_2_ and CaCl_2_ (Figure 6B). Human serum exposure is also known to inhibit the activity of many AMPs, compromising their therapeutic potential in vivo. The effect of human serum on the Lynronnes derivatives was therefore tested. After incubation with 50% human serum for 4 h, MICs were determined for all peptides. Against *S. aureus* , most derivatives retained activity comparable with that observed in MH medium, although scaffold‐dependent differences were observed. Lynronne‐2 derivatives were most affected: the MIC of Lynronne‐2 increased fourfold, whereas Lynronne‐2R lost all detectable activity (MIC increasing from 31.25 to > 500 μg/mL) (Figure 6C). A similar but less pronounced effect was observed against *E. coli* , for which MICs generally doubled and, in some cases, increased up to four‐fold for Lynronne‐3 derivatives. Despite this serum‐induced reduction in activity, the all‐Lys derivatives remained the most active compounds against *E. coli*, with even lower MIC values for Lynronne‐1K and Lynronne‐3K in the presence of serum (Figure 6D).

**FIGURE 6:**
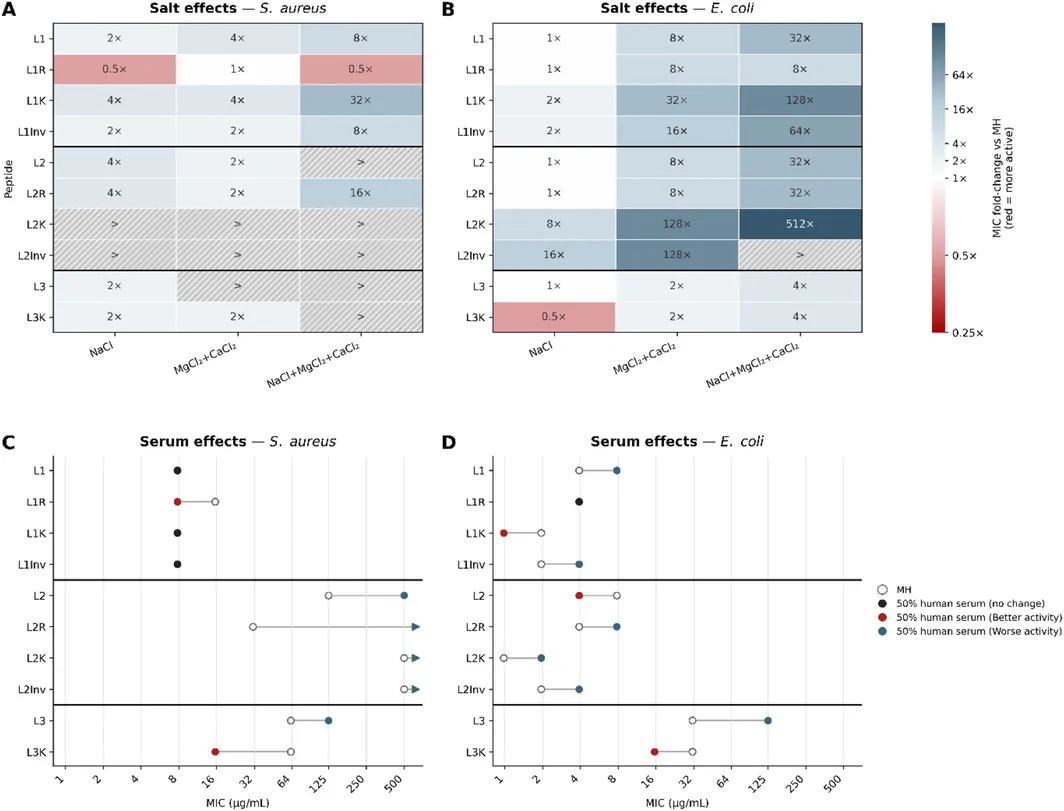
Effects of physiological salts and human serum on peptide antibacterial activity. In this figure, L1, L2, and L3 denote Lynronne‐1, Lynronne‐2, and Lynronne‐3, respectively; derivative labels follow the same convention (e.g., L1R denotes Lynronne‐1R). Panels A and B show MIC fold‐change in the presence of salt for *S. aureus* and *E. coli* , respectively. Red indicates increased activity, white indicates no change, and blue indicates reduced activity. Panels C and D compare MICs in the absence or presence of 50% human serum. MIC determination was performed in independent triplicates (*n* = 3).

### 3.4 Cytotoxicity

The toxicity of the Lynronnes derivatives was evaluated by cytotoxicity assays on four human cell types and hemolysis assays on human erythrocytes. Dose‐dependent curves used to determine CC_50_ are shown in Figure S14. Cytotoxicity was measured with the resazurin assay on lung (BEAS‐2B), liver (HepG2), skin (HaCaT), and intestinal (Caco‐2) cells to assess selectivity across different tissue contexts. Consistent with previous reports [31, 32, 46, 47], parental Lynronne‐1, Lynronne‐2, and Lynronne‐3 showed limited cytotoxicity, with cytotoxic concentration 50% (CC_50_) values ranging from 153 to 517 μg/mL for Lynronne‐1, 604 to > 1000 μg/mL for Lynronne‐2, and 560 to > 1000 μg/mL for Lynronne‐3 (Figures 7 and 9).

**FIGURE 7:**
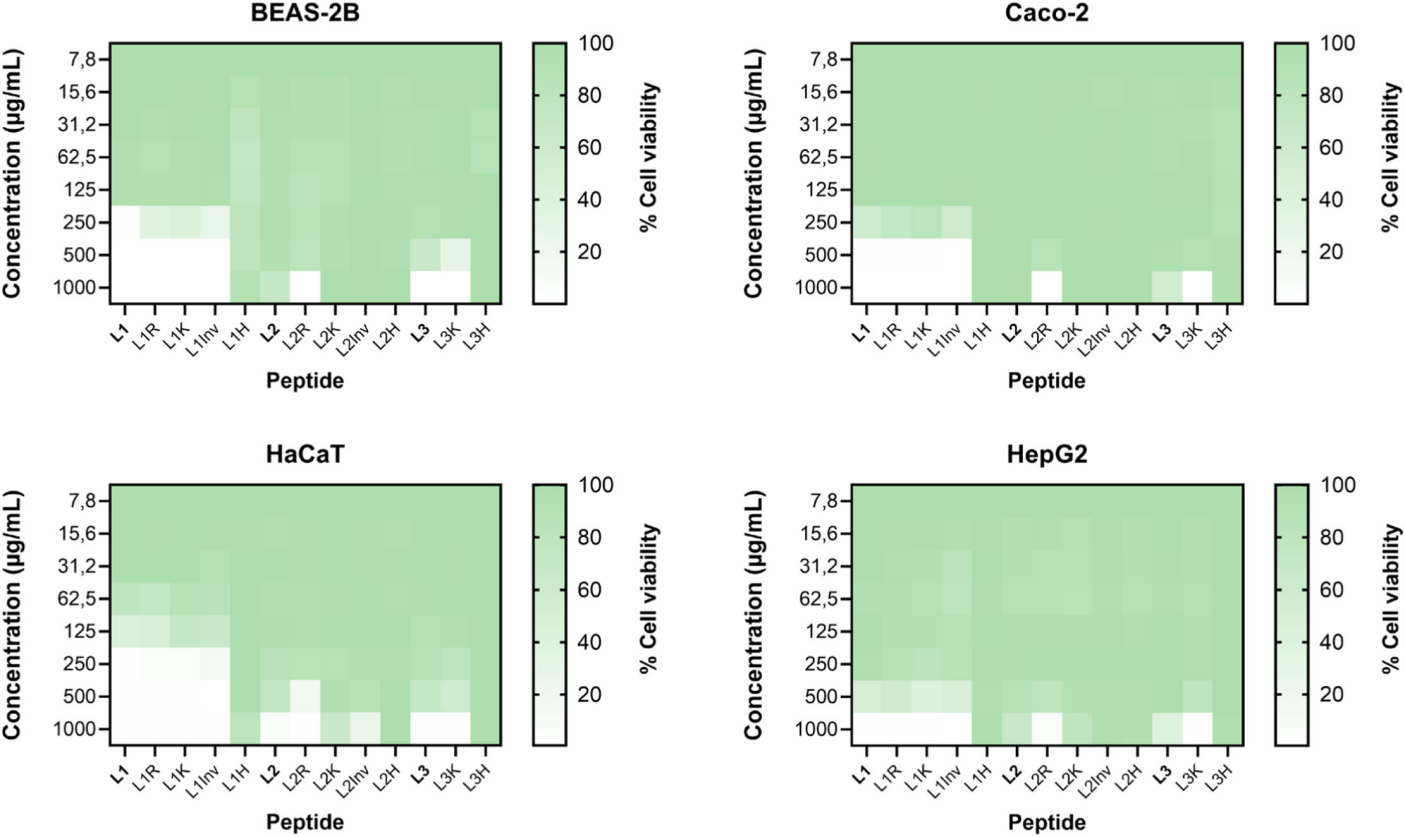
Cytotoxic activity of Lynronnes derivatives on human cells. In this figure, L1, L2, and L3 denote Lynronne‐1, Lynronne‐2, and Lynronne‐3, respectively; derivative labels follow the same convention (e.g., L1R denotes Lynronne‐1R). Cytotoxicity of the Lynronnes and their derivatives were tested after 48 h exposure of human lung (BEAS‐2B), intestinal (Caco‐2), skin (HaCaT), and liver (HepG2) cells to increase concentrations of peptides. Results are expressed as means of the percentage of cell viability (*n* = 3).

For Lynronne‐1, all derivatives showed reduced or unchanged cytotoxicity relative to the parental peptide. For Lynronne‐2, the all‐Arg variant showed increased toxicity, whereas for Lynronne‐3, the all‐Lys variant was more toxic than its parental counterpart (Figure 10). All histidine‐containing variants were nontoxic up to the highest concentration tested (> 1000 μg/mL).

Selectivity and safety of the peptides were further evaluated through measurement of their hemolytic activity (Figures 8 and 9). Dose‐dependent curves used to determine HC_50_ are shown in Figure S15. HC_50_ (the concentrations of peptides causing 50% hemolysis) values for all 13 peptides are presented in Figure 9. Overall, parental Lynronne‐1 and Lynronne‐3 and their derivatives were found to be more hemolytic compared with Lynronne‐2 and its derivatives. All‐Arg derivatives tended to exhibit higher hemolytic activity than their all‐Lys or all‐His counterparts (Figure 10).

**FIGURE 8:**
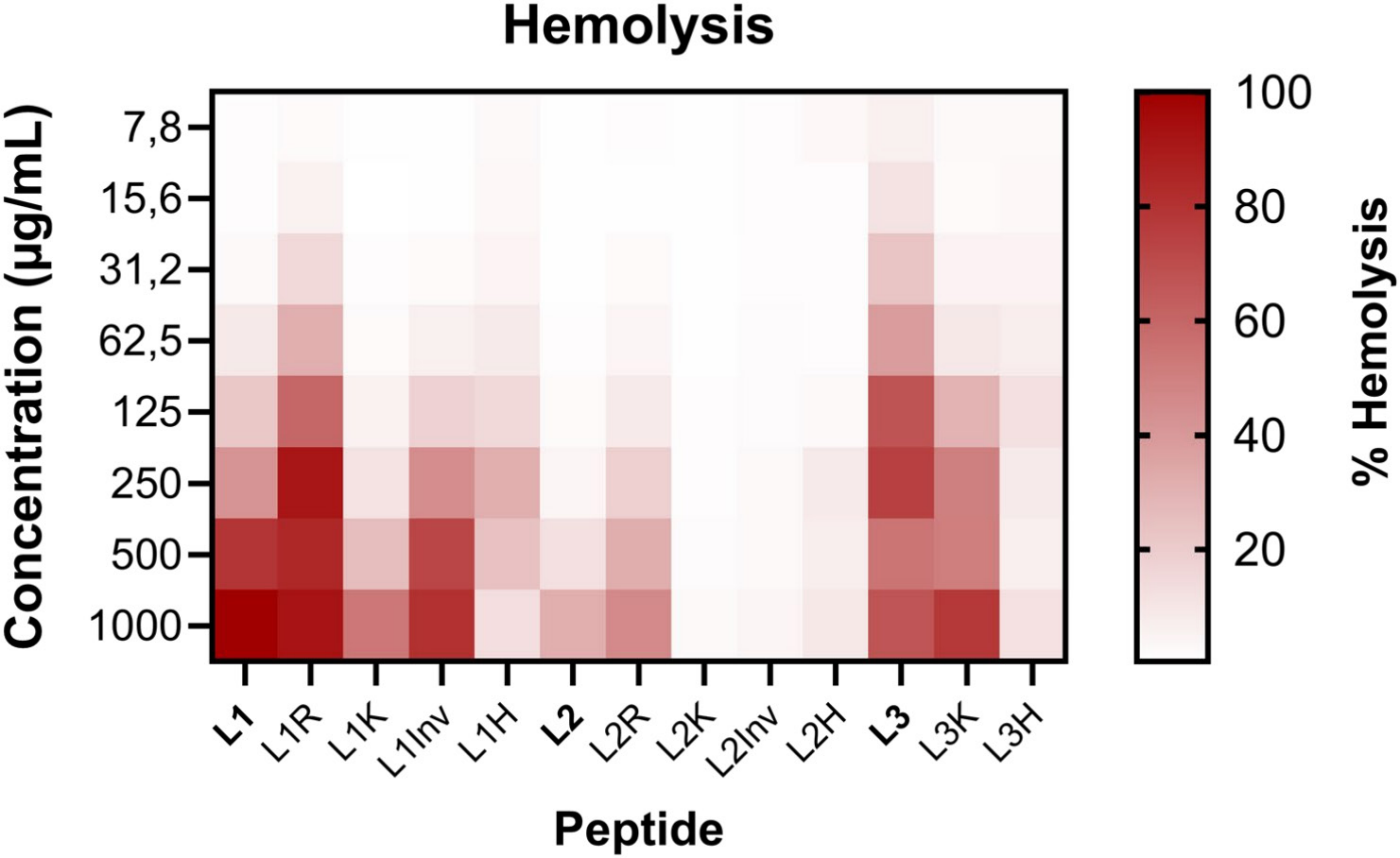
Hemolytic activity of Lynronnes derivatives on human red blood cells. In this figure, L1, L2, and L3 denote Lynronne‐1, Lynronne‐2, and Lynronne‐3, respectively; derivative labels follow the same convention (e.g., L1R denotes Lynronne‐1R). Human red blood cells were exposed for 1 h to increasing concentrations of peptides before determination of the release of hemoglobin. Results are expressed as percentage of hemolysis (means of *n* = 3) with Triton X‐100 used as positive control causing 100% cell lysis.

**FIGURE 9:**
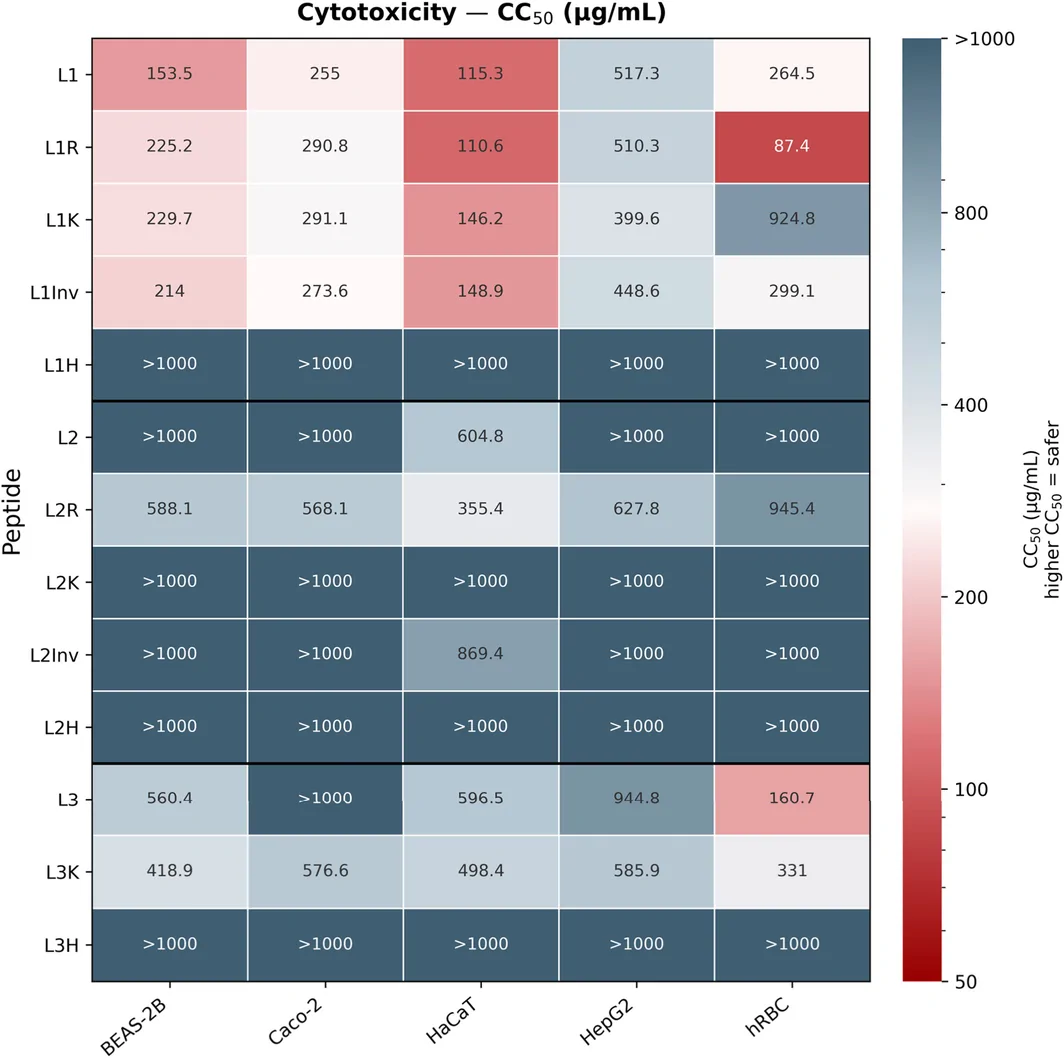
Evaluation of the innocuity of the Lynronnes derivatives. In this figure, L1, L2, and L3 denote Lynronne‐1, Lynronne‐2, and Lynronne‐3, respectively; derivative labels follow the same convention (e.g., L1R denotes Lynronne‐1R). Dark red indicates higher toxicity (lower CC_50_ or HC_50_ values), whereas blue indicates lower toxicity (higher CC_50_ or HC_50_ values), and deep blue color indicated CC50 > 1000 μg/mL. Values were determined from independent triplicates (*n* = 3).

**FIGURE 10:**
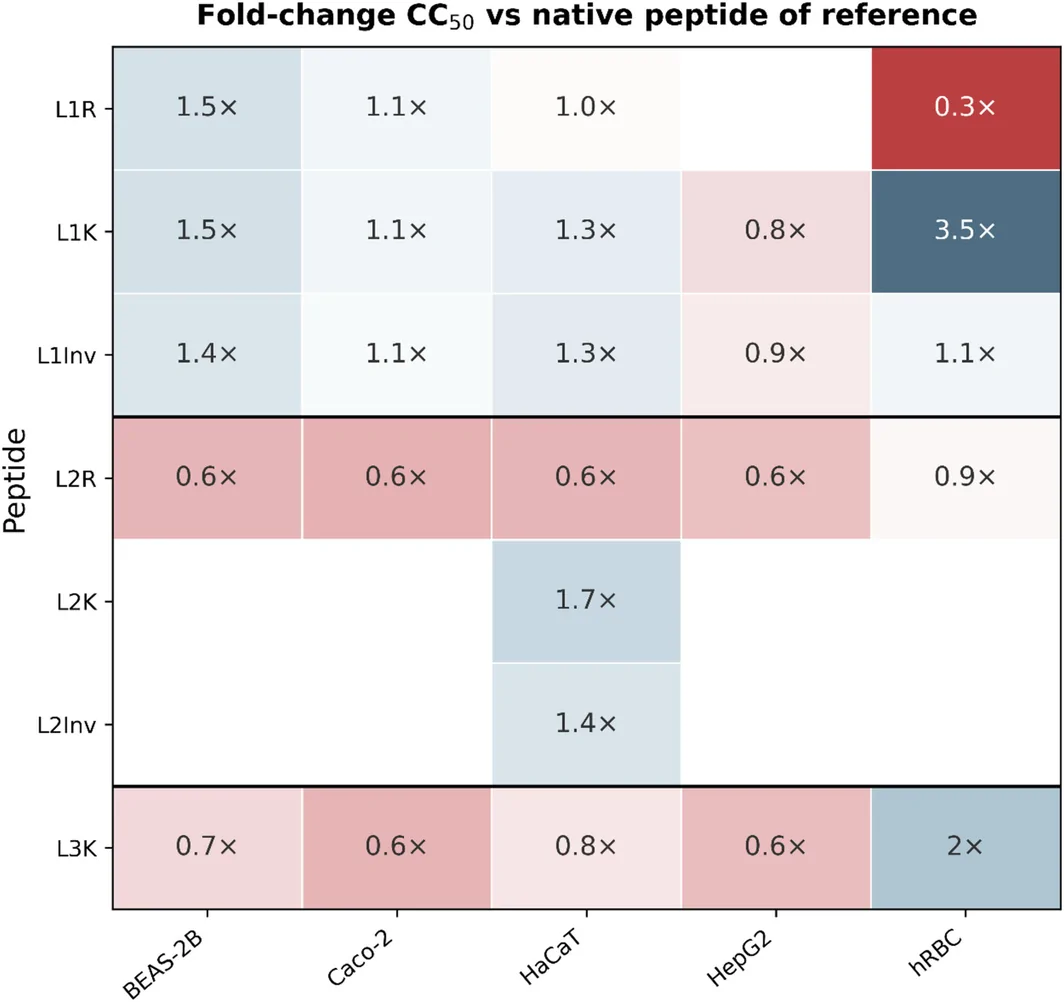
Fold changes in the toxicity of each derivative compared with the corresponding parental peptide. In this figure, L1, L2, and L3 denote Lynronne‐1, Lynronne‐2, and Lynronne‐3, respectively; derivative labels follow the same convention (e.g., L1R denotes Lynronne‐1R). Each derivative was compared with its corresponding parental peptide. Red color indicates increased toxicity compared with the parental peptide; blue color indicates decreased toxicity compared with the parental peptide. White color indicates a CC_50_ or HC_50_ equal to that of the corresponding parental peptide. Values were determined from independent triplicates (*n* = 3).

Selectivity indices (SIs) were calculated as a range for *S. aureus* (Gram‐positive) and *E. coli* (Gram‐negative), using the lowest CC_50_ or HC_50_ combined with the highest MIC as the conservative estimate, and the highest CC_50_ or HC_50_ combined with the lowest MIC as the favorable estimate (Figure 11). Several derivatives showed improved therapeutic profiles relative to the parental peptides. Whereas all‐Arg derivatives displayed ranges of SI comparable to the parental peptides, all‐Lys derivatives were found more selective with higher SI, except for Lynronne‐3 and its all‐Lys derivative where similar SI values were obtained.

**FIGURE 11:**
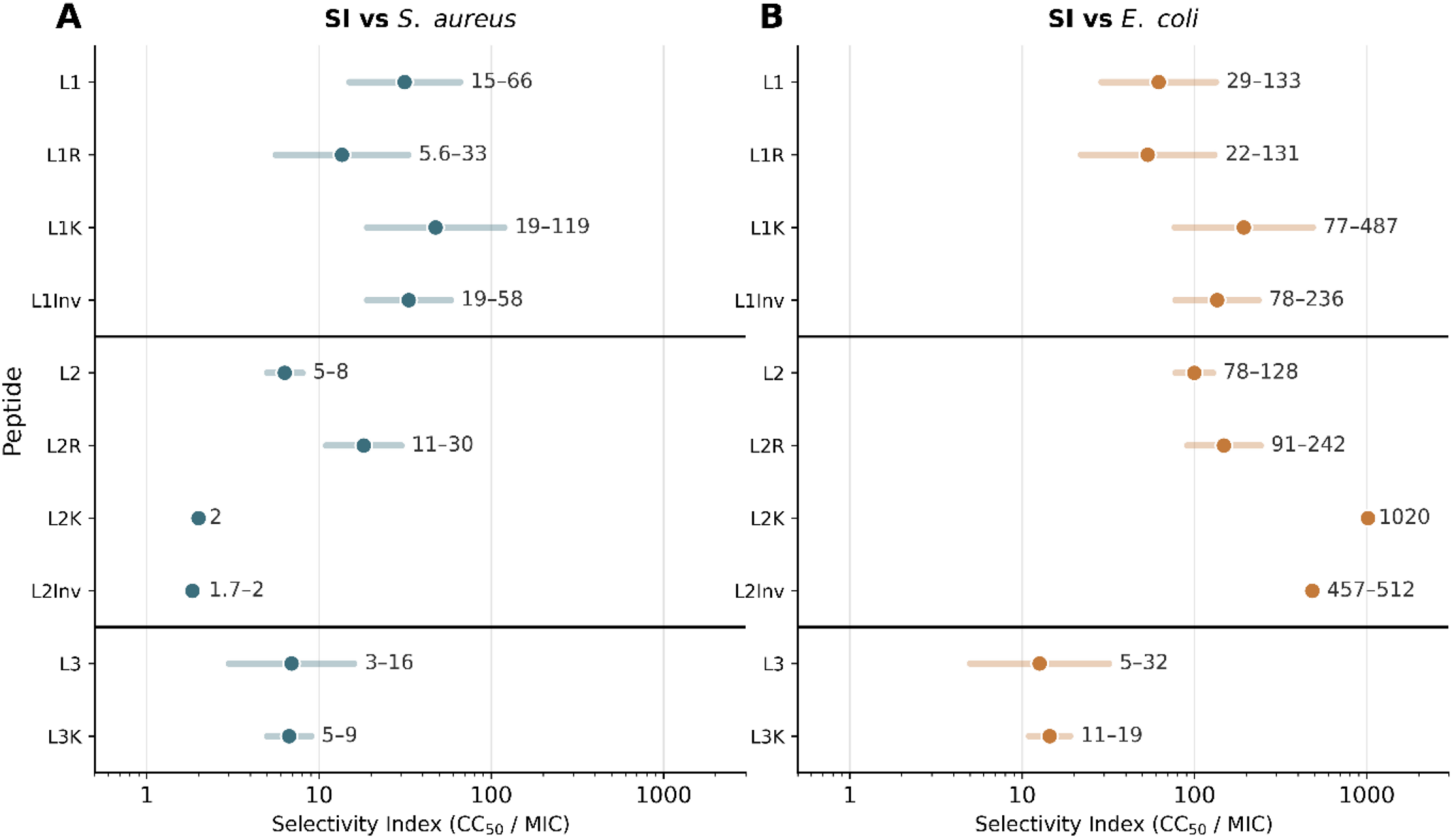
Selectivity index (SI) of the peptides. In this figure, L1, L2, and L3 denote Lynronne‐1, Lynronne‐2, and Lynronne‐3, respectively; derivative labels follow the same convention (e.g., L1R denotes Lynronne‐1R). The selectivity indexes (SI) of the parental peptides and their derivatives were calculated by dividing their CC_50_ or HC_50_ values by their MIC values obtained on *S. aureus* (ATCC 6538) or *E. coli* (ATCC 8739). Markers show the geometric means (√ (min × max)) of the SI range; bars span the observed min–max. Higher SI indicates higher selectivity. Results corresponding to independent triplicate (*n* = 3).

### 3.5 Mechanism of Actions

Previous studies have shown that parental Lynronnes cause antibacterial effect through permeabilization of the bacteria membrane [32]. To confirm that the Lynronnes derivatives also act through membrane permeabilization, we evaluated their impact on membrane integrity in *E. coli* and *S. aureus* by measuring ATP release using a luciferin/luciferase bioluminescence assay (Figure 12). For *S. aureus* , all peptides induced very rapid membrane lysis, with 100% ATP release reached within the first minute of incubation. Only Lynronne‐2R and Lynronne‐2K acted slowly, reaching maximal membrane permeabilization after 3 min of exposure. In the case of *E. coli* , membrane permeabilization was slower and less efficient. For all Lynronnes derivatives, except Lynronne‐2K and Lynronne‐2Inv, maximum permeabilization reached 60%–70% of the positive control within 20 min, consistent with a pore‐forming mechanism causing partial membrane disruption. Lynronne‐2K and Lynronne‐2Inv showed slower kinetics, requiring approximately 60 min to reach similar level of ATP release (Figure 12).

**FIGURE 12:**
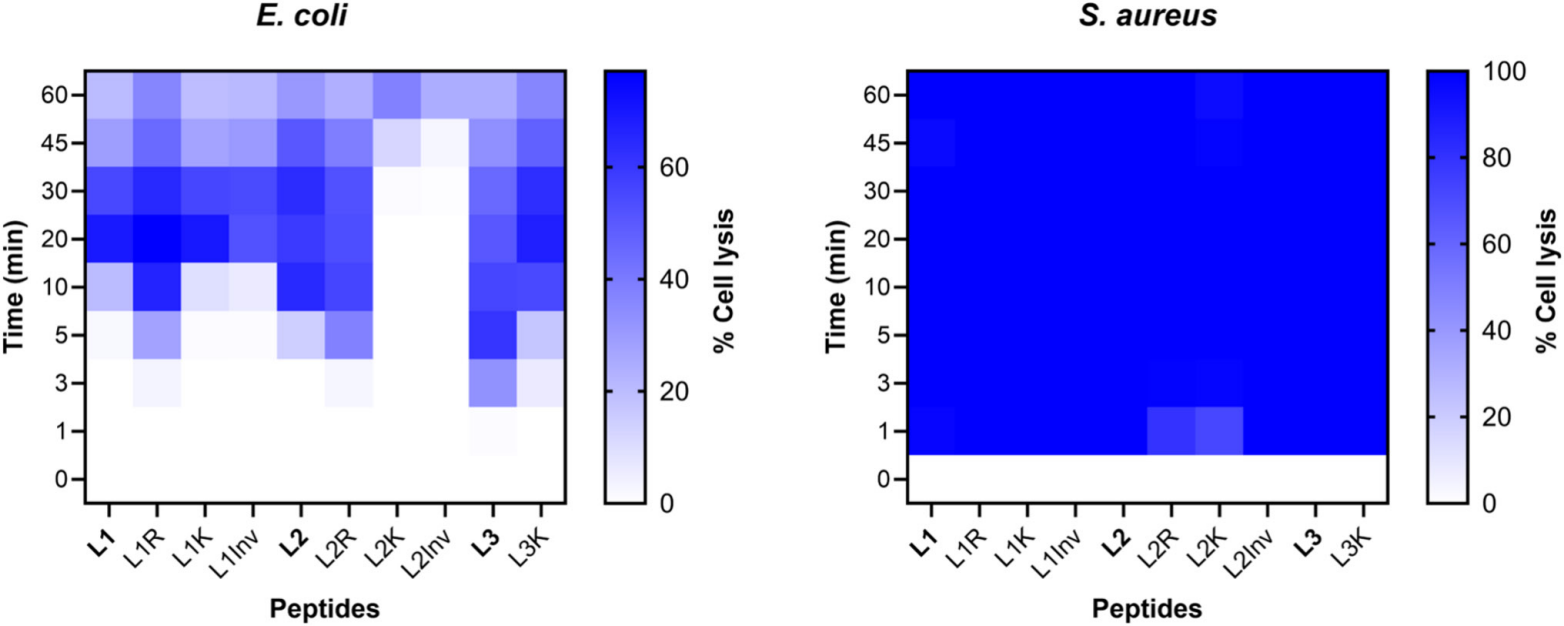
Membrane permeabilization of *E. coli* (ATCC 8739) (left) and *S. aureus* (ATCC 6538) (right) by Lynronnes peptides. In this figure, L1, L2, and L3 denote Lynronne‐1, Lynronne‐2, and Lynronne‐3, respectively; derivative labels follow the same convention (e.g., L1R denotes Lynronne‐1R). Time‐dependent bacterial membrane permeabilization caused by peptides at 4× MIC was measured through determination of the release of intracellular ATP by luminescence. Assay was performed in independent triplicate (*n* = 3).

### 3.6 Antibiofilm Activity of the Lynronnes Derivatives

The capacity of Lynronnes derivatives to inhibit biofilm formation and to eradicate preformed biofilms was measured using *S. aureus* (ATCC 6538) and the adherent‐invasive *E. coli* strain LF82, a well‐characterized biofilm former, as models. Biofilm biomass was quantified via a crystal violet assay. To better reflect the growth conditions encountered in biofilms (i.e., reduced growth rate and higher cell density), we first determined a modified MIC (MIC*) at an initial OD₆₀₀ of 0.05. As expected, the MIC* values were higher than the standard MICs, with increases ranging from 2‐fold to 32‐fold depending on the peptide. In addition to the all‐His derivatives, the Lynronne‐2K and Lynronne‐2Inv peptides, which showed no activity against planktonic *S. aureus*, were excluded from the subsequent biofilm assays with this strain (Table 3).

**TABLE 3:** Antibacterial activities of the Lynronnes derivatives against *S. aureus* (ATCC 6538) and *E. coli* LF82 in biofilm condition. MIC* corresponding to the MIC values measured in the conditions of biofilm assay are expressed in μg/mL. Results correspond to independent triplicates (*n* = 3).

Peptide	MIC* (μg/mL)	
	*S. aureus*	*E. coli* LF82
Lynronne‐1	15.62	15.62
Lynronne‐1R	15.62	15.62
Lynronne‐1K	15.62	7.81
Lynronne‐1Inv	7.81	31.25
Lynronne‐1H	> 500	> 500
Lynronne‐2	250	31.25
Lynronne‐2R	62.5	15.62
Lynronne‐2K	> 500	31.25
Lynronne‐2Inv	> 500	31.25
Lynronne‐2H	> 500	> 500
Lynronne‐3	125	62.5
Lynronne‐3K	125	31.25
Lynronne‐3H	> 500	> 500

All active peptides prevented biofilm formation at concentrations as low as 0.125× MIC* for both *S. aureus* and *E. coli* . For *S. aureus* , Lynronne‐3 and Lynronne‐3K exhibited the strongest preventive effect, reducing biofilm biomass by 85%, whereas Lynronne‐2 achieved only 38% inhibition under the same conditions. Against *E. coli* , Lynronne‐2K was the most effective, with a 65% reduction in biomass at 0.125× MIC* (Figure 13).

**FIGURE 13:**
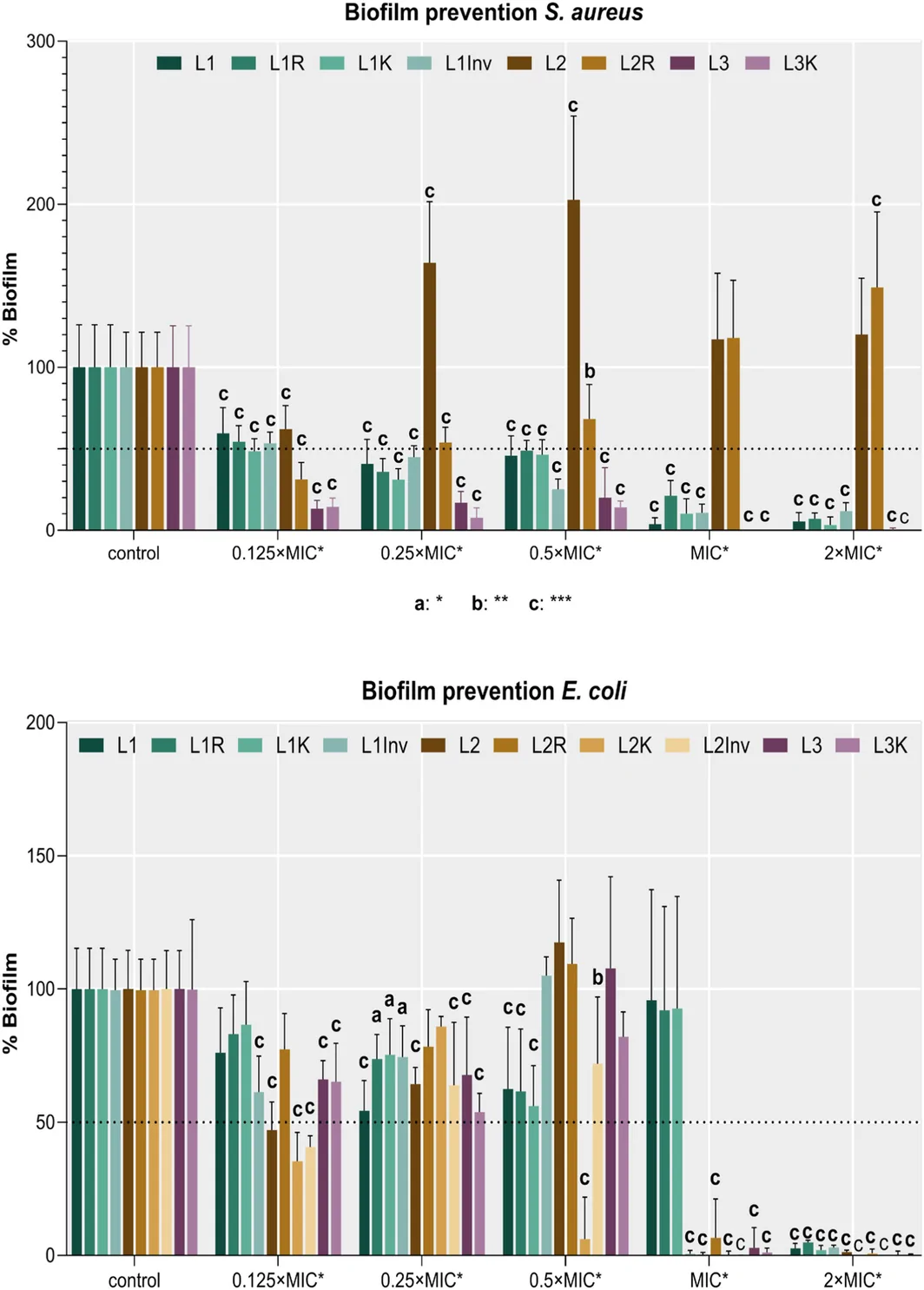
Effect of peptides on biofilm formation by *S. aureus* (ATCC 6538) and *E. coli* LF82. In this figure, L1, L2, and L3 denote Lynronne‐1, Lynronne‐2, and Lynronne‐3, respectively; derivative labels follow the same convention (e.g., L1R denotes Lynronne‐1R). Biofilm formation was quantified by crystal violet staining and expressed as a percentage of the corresponding untreated control, set to 100%. Data are presented as mean ± SD from three independent experiments (*n* = 3). Statistical comparisons were performed using two‐way analysis of variance (ANOVA) followed by Dunnett's multiple‐comparison test. Each peptide concentration was compared with the corresponding untreated control. (a) *p* < 0.05; (b) *p* < 0.01; (c) *p* < 0.001.

The antibiofilm activity was concentration‐dependent but non‐monotonic in both strains. This was particularly evident for Lynronne‐2 and Lynronne‐2R against *S. aureus* , where biomass reached up to 200% of the untreated control at 0.5× MIC* for Lynronne‐2. In *E. coli* , Lynronne‐2K reduced biofilm formation by 65% at 0.125× MIC*, but increasing the concentration to 0.25× MIC* unexpectedly lowered the inhibition to just 14%, leaving a substantially greater biofilm biomass than at the lower concentration. Overall, this biphasic profile suggests that for certain peptides, specific sub‐MIC* concentrations may paradoxically promote biofilm formation, whereas lower concentrations remain effectively inhibitory (Figure 13).

Eradication of established biofilms required higher peptide concentration than prevention, with marked differences in efficacy between peptides and bacterial strains. All active peptides reduced preformed biofilms at concentrations ranging from MIC* to 4× MIC* for both strains. Lynronne‐3 was the most effective overall, reducing biofilm biomass by 92% and 74% at 4× MIC*, and by 54% and 52% at MIC*, for *S. aureus* and *E. coli* , respectively. Against *S. aureus* , Lynronne‐2 and Lynronne‐2R displayed the weakest activity, achieving only 30% reduction at 4× MIC*. For *E. coli* , Lynronne‐1K was the least potent, with reductions of only 44% and 8% at 4× MIC* and MIC*, respectively (Figure 14).

**FIGURE 14:**
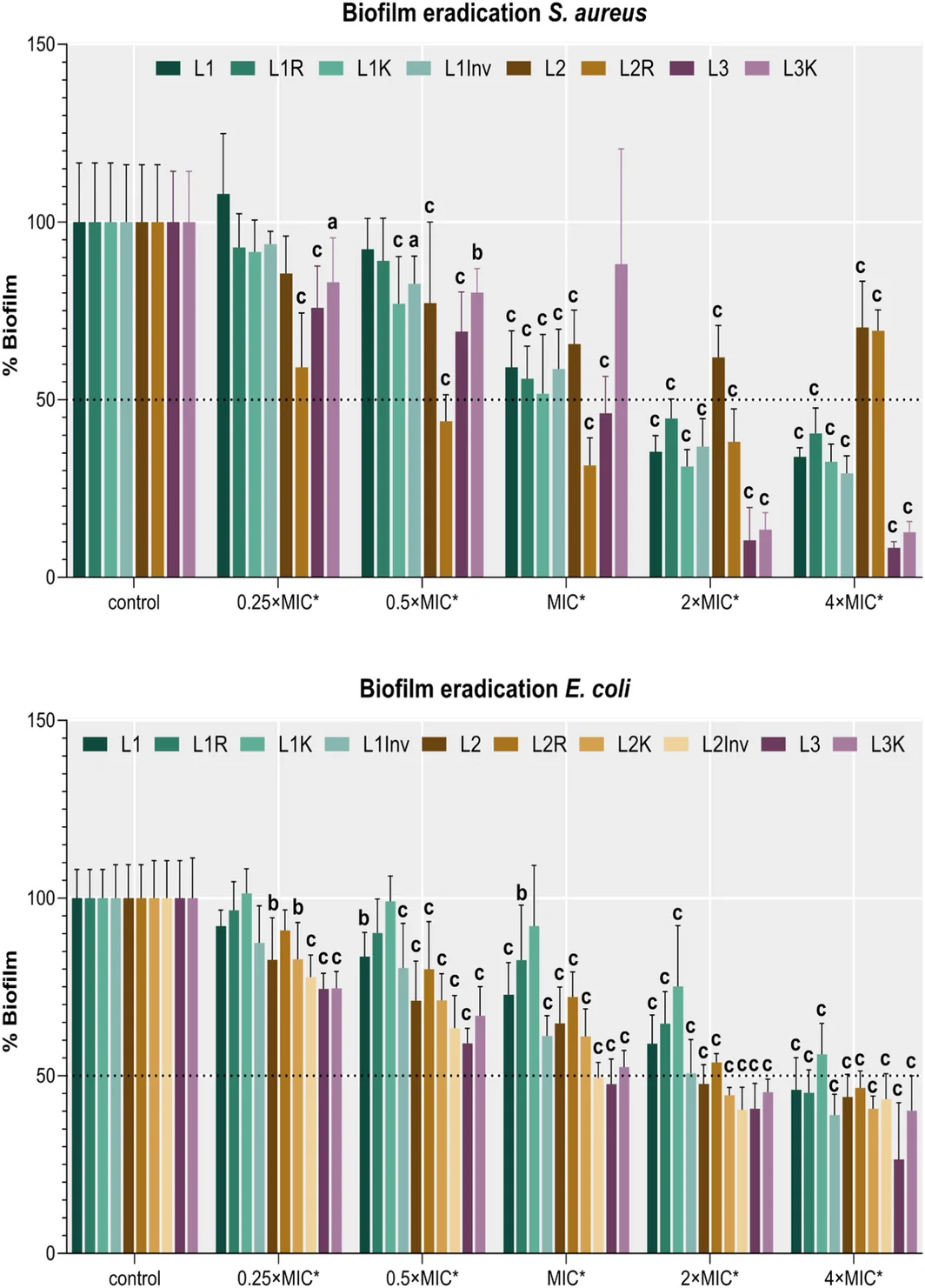
Effect of peptides on preformed biofilms of *S. aureus* (ATCC 6538) and *E. coli* LF82. In this figure, L1, L2, and L3 denote Lynronne‐1, Lynronne‐2, and Lynronne‐3, respectively; derivative labels follow the same convention (e.g., L1R denotes Lynronne‐1R). Biofilm biomass was quantified by crystal violet staining and expressed as a percentage of the corresponding untreated control, set to 100%. Data are presented as mean ± SD from three independent experiments (*n* = 3). Statistical comparisons were performed using two‐way analysis of variance (ANOVA) followed by Dunnett's multiple‐comparison test. Each peptide concentration was compared with the corresponding untreated control. (a) *p* < 0.05; (b) *p* < 0.01; (c) *p* < 0.001.

## 4 Discussion

Since their discovery, antibiotics have saved millions of lives and revolutionized medicine. However, the resurgence of multidrug‐resistant bacteria threatens this golden age [52], reigniting interest in alternative strategies such as AMPs. AMPs are promising candidates due to their ability to kill both Gram‐positive and Gram‐negative bacteria, a key feature of many AMPs being their cationic character, which promotes electrostatic interactions with negatively charged bacterial membranes allowing them to selectively disrupt their membrane. In this study, we generated derivatives of the previously described Lynronne‐1, Lynronne‐2, and Lynronne‐3 peptides [31, 32], by systematically varying the cationic residues (Arg, Lys, or His) because such substitutions may affect their structure and their activity. A total of 13 peptides were designed and synthesized.

Analysis of secondary structure by CD spectroscopy revealed that all Lynronnes derivatives are disordered in aqueous buffer but adopt a typical α‐helical conformation in the presence of SDS micelles [53] (membrane‐mimetic environment). This behavior is consistent with previous reports for Lynronne‐1 and Lynronne‐2 and supports a model in which these peptides fold upon membrane interaction. Substituting native cationic residues (Arg, Lys, His) with other basic amino acids influenced the helical content, though no universal trend emerged across the three scaffolds. For example, the reverse derivative Lynronne‐1Inv showed reduced helicity (47%) compared with parental Lynronne‐1 (80%) but retained or slightly improved antimicrobial activity, with decreased toxicity. Similarly, Lynronne‐2R (all‐Arg) decreased from 77% to 43% helix. Lynronne‐3 exhibited the lowest helical content (34%) and the lowest antimicrobial activity (MIC = 31.25 μg/mL against *E. coli* ), suggesting that a minimum helical propensity may contribute to activity. However, it is well known that the relationship between helicity and antimicrobial activity is not straightforward [54]. Lynronne‐1Inv, for example, demonstrates that reduced α‐helix content does not necessarily impair activity, suggesting that other factors, such as charge distribution, hydrophobicity, or amphipathicity, may compensate. Overall, although the nature of cationic residues can modulate α‐helical propensity, helicity alone does not predict antimicrobial potency, and the observed effects are scaffold‐dependent [55].

We next assessed the antimicrobial activity of the 13 Lynronnes derivatives against a panel of eight ESKAPEE strains. The all‐histidine variants were inactive up to 500 μg/mL, consistent with the loss of this residue's cationic charge at physiological pH [29, 56]. In contrast, the other derivatives showed variable activity depending on both the bacterial strain and the scaffold. *Escherichia coli* was the most sensitive species, with the all‐Lys variants (Lynronne‐1K, Lynronne‐2K, Lynronne‐3K) showing the greatest potency. Notably, Lynronne‐2K exhibited an 8‐fold improvement in MIC (0.98 μg/mL) compared with parental Lynronne‐2 (7.81 μg/mL) against *E. coli* ATCC 8739. Against Gram‐positive bacteria, the all‐Arg variants (e.g., Lynronne‐2R) tended to be more active than their Lys‐substituted counterparts. Overall, and as has been shown in previous studies, the impact of substituting cationic residues was strain‐ and scaffold‐dependent and no universal rule emerged [29, 55, 57, 58, 59]. Given the clinical importance of *E. coli* , we tested the derivatives against four pathogenic or resistant isolates: enteropathogenic *E. coli* (EPEC) and enterohemorrhagic *E. coli* (EHEC) that are associated with lethal diarrhea [35], the adherent‐invasive *E. coli* reference strain LF82, a strain associated with intestinal inflammation and Crohn's disease [60, 61], and the ESBL producer amoxicillin‐resistant uropathogenic isolate (UPEC) CF1702. The all‐Lys variants again showed excellent activity, with Lynronne‐1K achieving an MIC of 0.98 μg/mL against all four strains, matching the activity observed against the reference strain *E. coli* ATCC 8739. This suggests that Lys‐rich variants may be particularly favorable for targeting *E. coli* , including pathogenic and/or antibiotic‐resistant isolates.

Beyond their potential as alternatives to conventional antibiotics, AMPs may also be promising candidates for developing medical countermeasures against bacterial agents of biodefense relevance. We therefore evaluated the activity of Lynronnes derivatives against selected bacterial agents requiring BSL‐3 containment, including *Bacillus anthracis* , *Burkholderia mallei* , *Burkholderia pseudomallei* , *Brucella melitensis* , *Francisella tularensis* , and *Yersinia pestis* . Compared with the results obtained against *E. coli* , antimicrobial activity was more restricted and strongly species‐specific. This limited activity, particularly against Gram‐negative agents, may partly reflect pathogen‐specific cell‐envelope adaptations that reduce susceptibility to cationic AMPs, including LPS/lipid A remodeling, altered surface charge, and reduced outer‐membrane permeability [62, 63]. Such resistance‐associated envelope features have been described in several of the pathogens tested here, including *Burkholderia* spp., *Brucella* spp., *Francisella* spp., and *Y. pestis* [64, 65, 66, 67]. Consistent with this, the most potent activity was observed against *B. anthracis* , particularly for Lynronne‐1‐derived peptides, with MICs of 31.2 μg/mL for Lynronne‐1, Lynronne‐1R, Lynronne‐1K, and Lynronne‐1Inv. In contrast, no activity was detected against *B. mallei* or *B. pseudomallei* at the highest concentration tested, highlighting the limited efficacy of Lynronnes derivatives against these highly resistant Gram‐negative pathogens. Activity against *B. melitensis* , *F. tularensis* , and *Y. pestis* was limited to selected compounds, among which Lynronne‐1R showed the broadest profile, with MICs of 62.5 μg/mL against *B. melitensis* and 250 μg/mL against *F. tularensis* and *Y. pestis* . Overall, these data indicate that Lynronnes derivatives retain activity against selected bacterial biothreat agents.

With the three mycobacterial pathogenic strains, all‐Arg derivatives showed enhanced antimicrobial potency, particularly against *M. tuberculosis* mc^2^6230, while all‐Lys peptides retained partial activity against *M. abscessus* and *M. marinum* . In contrast, all‐His and reverse (Inv) peptides showed consistently diminished or abolished activity, highlighting the critical role of cationic residue identity in modulating efficacy across mycobacterial species.

Following the broad‐spectrum screening, *E. coli* and *S. aureus* were selected as representative Gram‐negative and Gram‐positive model strains, respectively, for subsequent in‐depth characterization of Lynronnes derivatives. These strains were used to assess peptide performance under physiologically relevant conditions, including salt and human serum exposure, and to investigate membrane permeabilization and antibiofilm activity. The therapeutic utility of AMPs is often constrained by loss of activity under physiological conditions, including high salt concentrations and the presence of serum proteins [68]. Our results confirm that salt tolerance is highly dependent on both the nature of the cationic residues and the peptide scaffold. Arg‐rich variants generally exhibited superior salt tolerance compared with their Lys‐substituted counterparts, consistent with the ability of the guanidinium group of arginine to form stronger electrostatic interactions with membrane phosphates. However, this trend was not universal, as the all‐Lys variant of Lynronne‐3 (Lynronne‐3K) displayed better salt tolerance than its all‐Arg peptide, underscoring the scaffold‐dependent nature of this property [69, 70]. Regarding serum stability, all derivatives retained appreciable activity against *S. aureus* , although Lynronne‐2 variants were the most affected, with the all‐Arg peptide (Lynronne‐2R) losing all detectable activity at > 500 μg/mL. This susceptibility may reflect increased proteolytic degradation or enhanced serum protein binding, both of which are commonly associated with all‐Arg peptides. Against *E. coli* , serum‐induced activity loss was generally less pronounced, with MICs increasing only twofold to fourfold. Notably, despite this reduction, the all‐Lys derivatives (Lynronne‐1K, Lynronne‐2K, Lynronne‐3K) consistently remained the most active compounds against *E. coli* under serum conditions, reinforcing their potential for further development.

Good selectivity toward bacteria over eukaryotic/human cells is essential for estimating the therapeutic potential of AMPs. Overall, most Lynronne‐1 derivatives showed improved selectivity against *E. coli* and *S. aureus* compared with the parental peptide, due to decreased cytotoxicity (e.g., Lynronne‐1K, CC_50_ = 146–925 μg/mL vs. Lynronne‐1, CC_50_ = 115–517 μg/mL) (depending on the cell type). For Lynronne‐2, the all‐Arg variant (Lynronne‐2R) was more toxic than the parental peptide, reducing its selectivity, whereas other derivatives showed comparable or improved profiles. In contrast, for Lynronne‐3, the all‐Lys variant (Lynronne‐3K) exhibited increased toxicity, but this did not translate into improved selectivity. The all‐His variants were nontoxic up to 1000 μg/mL, but their nontoxicity is limited by their lack of antimicrobial activity. Consistent with previous reports, Arg‐substituted variants tended to exhibit higher hemolytic activity than their Lys‐substituted counterparts [29, 71, 72]. SIs (SI = HC_50_/MIC) ranged from 11 to >1020, with the best profiles observed for Lynronne‐2K (SI > 1020 against *E. coli* ), and from 1.7 to 119, with the best profiles observed for Lynronne‐1K (SI = 19–119 against *S. aureus* ).

Beyond the nature and number of cationic residues, the balance between cationic charge and hydrophobicity is known to be a major determinant of both antimicrobial efficacy and cytotoxicity toward eukaryotic cells [23]. An optimal amphiphilic balance allows the peptides to bind to anionic bacterial membranes via electrostatic interactions while retaining sufficient hydrophobicity to insert into the membrane core [73]. However, excessive hydrophobicity promotes nonselective interactions with zwitterionic eukaryotic membranes, increasing hemolysis and cytotoxicity, whereas insufficient hydrophobicity impairs membrane insertion and reduces antibacterial activity. This principle has been studied in the context of AMPs polymers mimics, where the balance between cationic and hydrophobic domains can be systematically tuned by varying the nature and number of cationic groups or the length and density of hydrophobic side chains [74]. Studies on amphiphilic polymethacrylates, poly(α‐amino acid) copolymers, and polyphosphoniums have consistently shown that antibacterial activity and hemolytic toxicity are governed by the same structural parameters, such that increasing hydrophobicity simultaneously increases both activity and toxicity [75]. A similar trade‐off is observed with the Lynronne derivatives studied here: Variants composed entirely of arginine residues, which are more hydrophobic than their lysine‐based counterparts, tended to exhibit higher hemolytic activity, consistent with the well‐established correlation between arginine content and the disruption of eukaryotic membranes. Conversely, variants composed entirely of histidine residues, which lose their cationic character at physiological pH, exhibited neither toxicity nor antimicrobial activity, thereby illustrating the need to co‐optimize charge and hydrophobicity rather than maximizing them independently. These observations align with the concept of “amphiphilic balance” a narrow compositional range that maximizes membrane selectivity, as described for both natural AMPs and their synthetic analogues [76].

Unlike many conventional antibiotics that target intracellular processes, AMPs generally act by disrupting the bacterial membrane, thereby inducing rapid cell death [77, 78]. This membrane‐centric mechanism is thought to explain the rarity of resistance against AMPs. Consistent with this model, all Lynronnes derivatives induced ATP leakage, confirming membrane permeabilization [79]. However, differences in kinetics were observed across strains and cationic substitutions. In *S. aureus* , all derivatives caused rapid and total membrane disruption within 1 min of contact, except for Lynronne‐2R and Lynronne‐2K, which required 3 min to reach this effect. In *E. coli* , membrane permeabilization was slower, with maximum ATP release observed after 3–10 min of exposure. The Lynronne‐2 variants Lynronne‐2K (all‐Lys) and Lynronne‐2Inv (reverse) showed a striking delay, requiring approximately 45 min to reach maximal ATP release. Remarkably, these two slow‐permeabilizing variants (Lynronne‐2K and Lynronne‐2Inv) also exhibited the strongest antimicrobial activity against *E. coli* (MICs of 0.98 and 1.95 μg/mL, respectively). Although a slower pore‐forming activity of AMPs in Gram‐negative than in Gram‐positive bacteria is classically observed [42], this intriguing dissociation between permeabilization kinetics and antibacterial potency suggests distinct mechanisms unrelated to membrane permeabilization as was previously speculated for the parental Lynronne‐2 [31].

Biofilms are surface‐attached bacterial communities embedded in a self‐produced matrix of extracellular polymeric substances, principally exopolysaccharides, extracellular DNA, and proteins. Their reduced susceptibility to antimicrobials is a major clinical concern, and numerous AMPs have been reported to act on both biofilm formation and established biofilms [33, 80, 81]. A striking dichotomy was observed in the peptides' antibiofilm activity. Eradication of mature biofilms was dose‐dependent in both *S. aureus* and *E. coli* . In contrast, inhibition of biofilm formation was non‐monotonic in both species: At intermediate sub‐MIC concentrations, biomass exceeded that measured at lower concentrations and, for Lynronne‐2 and Lynronne‐2R in *S. aureus* , exceeded the untreated control. Subinhibitory stimulation of biofilm formation has been documented for conventional antibiotics, including fluoroquinolones in *E. coli* and MRSA, and folate‐pathway inhibitors in *E. coli* [82, 83]. The linear profile observed in the eradication assay suggests that this biphasic effect is not an artifact but a genuine biological phenomenon. We hypothesize that this pro‐biofilm response is specific to the early adhesion phase, in which metabolically active bacterial cells perceive sub‐MIC concentrations as sublethal stress, triggering envelope stress signaling pathways and extracellular matrix overproduction. Sublethal AMP exposure has been shown to prime *E. coli* toward tolerance and persistence through matrix‐associated determinants [84]. In *P. aeruginosa* , sub‐MIC antibiotics induce biofilm formation through an OprF‐dependent pathway that raises intracellular c‐di‐GMP [85]; an analogous OmpA‐dependent route has been proposed in *E. coli* [85]. The observation of this biphasic effect in both *S. aureus* and *E. coli* suggests that it may be conserved across Gram‐positive and Gram‐negative bacteria, possibly involving distinct yet functionally analogous stress‐response pathways, a phenomenon described by previous studies [86, 87]. The non‐monotonic profile was not observed in mature biofilms, possibly because reduced metabolic activity limits inducible stress signaling, leaving direct membrane activity as the dominant mode of action. In this assay, all active peptides reduced preformed biofilms dose‐dependently, Lynronne‐3 being the most effective in both species. The contrast between the two assays indicates that the physiological state of the target population—actively adhering versus sessile and mature—is a primary determinant of the outcome of peptide exposure. Sub‐MIC exposure windows should therefore be characterized systematically during AMP development, as suboptimal concentrations may favor surface colonization rather than suppress it. The magnitude of this effect varied between peptides and strains, indicating that it is not a uniform response but a sequence‐ and strain‐dependent consideration that can be addressed through careful dose optimization and candidate selection [88, 89]. Our results underscore the importance of careful dose optimization when considering AMPs for clinical use, as concentrations that are too low could paradoxically favor persistence rather than suppress it. The magnitude of this effect varied between peptides and strains, suggesting that future therapeutic development should include systematic sub‐MIC profiling to identify and avoid pro‐biofilm concentration windows.

## 5 Conclusion

Although further in vivo studies are needed to evaluate it, overall, this study demonstrates that systematic substitution of cationic residues in Lynronnes peptides modulates their structure, antimicrobial activity, selectivity, and safety profile in a sequence‐dependent manner. The all‐Arg variants have improved antibacterial activity and salt tolerance, but this advantage was often counterbalanced by increased hemotoxicity, whereas the all‐Lys derivatives frequently provided the best balance between antibacterial potency and selectivity, particularly against *E. coli* . In contrast, the all‐His peptides lacked antimicrobial activity despite their absence of toxicity, underscoring that the loss of cationic charges at physiological pH compromises antimicrobial activity while limiting toxicity. Activity against several biothreat agents and mycobacteria, although modest, further demonstrates the potential of these peptides in medicine. The loss of potency under salt or serum conditions and the promotion of biofilm formation at subinhibitory concentrations identify important areas for optimization. Collectively, these findings establish a rational framework for the future design of Lynronnes‐derived peptides and identify Lys substitution as a particularly promising strategy for improving the balance between antimicrobial activity and safety.

## Funding

This study was supported by the Coordenação de Aperfeiçoamento de Pessoal de Nível Superior, No. Ph‐C1062/25.

## Conflicts of Interest

The authors declare no conflicts of interest.

## Supporting information


**Figure S1:** Analytical RP‐HPLC chromatogram (upper) and ESI‐MS spectrum (lower) of peptide Lynronne‐1.
**Figure S2:** Analytical RP‐HPLC chromatogram (upper) and ESI‐MS spectrum (lower) of peptide Lynronne‐1R.
**Figure S3:** Analytical RP‐HPLC chromatogram (upper) and ESI‐MS spectrum (lower) of peptide Lynronne‐1K.
**Figure S4:** Analytical RP‐HPLC chromatogram (upper) and ESI‐MS spectrum (lower) of peptide Lynronne‐1Inv.
**Figure S5:** Analytical RP‐HPLC chromatogram (upper) and ESI‐MS spectrum (lower) of peptide Lynronne‐1H.
**Figure S6:** Analytical RP‐HPLC chromatogram (upper) and ESI‐MS spectrum (lower) of peptide Lynronne‐2.
**Figure S7:** Analytical RP‐HPLC chromatogram (upper) and ESI‐MS spectrum (lower) of peptide Lynronne‐2R.
**Figure S8:** Analytical RP‐HPLC chromatogram (upper) and ESI‐MS spectrum (lower) of peptide Lynronne‐2K.
**Figure S9:** Analytical RP‐HPLC chromatogram (upper) and ESI‐MS spectrum (lower) of peptide Lynronne‐2Inv.
**Figure S10:** Analytical RP‐HPLC chromatogram (upper) and ESI‐MS spectrum (lower) of peptide Lynronne‐2H.
**Figure S11:** Analytical RP‐HPLC chromatogram (upper) and ESI‐MS spectrum (lower) of peptide Lynronne‐3.
**Figure S12:** Analytical RP‐HPLC chromatogram (upper) and ESI‐MS spectrum (lower) of peptide Lynronne‐3K.
**Figure S13:** Analytical RP‐HPLC chromatogram (upper) and ESI‐MS spectrum (lower) of peptide Lynronne‐3H.
**Figure S14:** Concentration–response curves for the cytotoxicity of Lynronnes peptides. In this figure, L1, L2, and L3 denote Lynronne‐1, Lynronne‐2, and Lynronne‐3, respectively; derivative labels follow the same convention (e.g., L1R denotes Lynronne‐1R). Cell viability is expressed as percentage of untreated control/percentage viability. Data are presented as mean ± SEM from *n* = 3 independent experiments. Curves were fitted using nonlinear regression in GraphPad Prism 11.1.0.
**Figure S15:** Concentration–response curves for the hemolytic activity of Lynronnes peptides against human erythrocytes. In this figure, L1, L2, and L3 denote Lynronne‐1, Lynronne‐2, and Lynronne‐3, respectively; derivative labels follow the same convention (e.g., L1R denotes Lynronne‐1R). Hemolysis was quantified as a percentage relative to the positive lysis control. Data are presented as mean ± SEM from *n* = 3 independent experiments. Curves were fitted using nonlinear regression in GraphPad Prism 11.1.0.
**Table S1:** Antibacterial activity of the Lynronnes derivatives against ESKAPEE bacteria. MIC values are expressed in μg/mL. In this table, L1, L2, and L3 denote Lynronne‐1, Lynronne‐2, and Lynronne‐3, respectively; derivative labels follow the same convention (e.g., L1R denotes Lynronne‐1R). Assay performed in *n* = 3.
**Table S2:** Antibacterial activity of the Lynronnes derivatives against *E. coli* pathogenic and resistant *E. coli* strains. MIC values are expressed in μg/mL. In this table, L1, L2, and L3 denote Lynronne‐1, Lynronne‐2, and Lynronne‐3, respectively; derivative labels follow the same convention (e.g., L1R denotes Lynronne‐1R). Assay performed in *n* = 3.
**Table S3:** Antibacterial activity of the Lynronnes derivatives against selected biothreat agents. MIC values are expressed in μg/mL. In this table, L1, L2, and L3 denote Lynronne‐1, Lynronne‐2, and Lynronne‐3, respectively; derivative labels follow the same convention (e.g., L1R denotes Lynronne‐1R). Assay performed in *n* = 3.
**Table S4:** Antibacterial activity of the Lynronnes derivatives against mycobacteria. MIC values are expressed in μg/mL. In this table, L1, L2, and L3 denote Lynronne‐1, Lynronne‐2, and Lynronne‐3, respectively; derivative labels follow the same convention (e.g., L1R denotes Lynronne‐1R). Assay performed in *n* = 3.
**Table S5:** Effect of salt on the antibacterial activity of the Lynronnes derivatives against *E. coli* (ATCC 8739) and *S. aureus* (ATCC 6538). MICs were measured in the presence of physiological salt concentrations (NaCl, 150 mM; MgCl2, 1 mM; CaCl2, 2 mM). MIC are expressed in μg/mL. In this table, L1, L2, and L3 denote Lynronne‐1, Lynronne‐2, and Lynronne‐3, respectively; derivative labels follow the same convention (e.g., L1R denotes Lynronne‐1R). Assay performed in *n* = 3.
**Table S6:** Antibacterial activities of the Lynronnes derivatives against *E. coli* (ATCC 8739) and *S. aureus* (ATCC 6538) in the presence of human serum (50%, 4 h incubation). In this table, L1, L2, and L3 denote Lynronne‐1, Lynronne‐2, and Lynronne‐3, respectively; derivative labels follow the same convention (e.g., L1R denotes Lynronne‐1R). MIC are expressed in μg/mL.
