# Supplementary material for: Influence of the Nature of the Cationic Residues on the Activity of the Antimicrobial Peptides of the Lynronne Family
Source: J Pept Sci. 2026 Nov 3;32(12):e70146. doi: 10.1002/psc.70146 (PMC13731755; doi:10.1002/psc.70146)
Supplement: Supplementary file 1 [file PSC-32-e70146-s001.docx]

# Influence of the nature of the cationic residues on the activity of the antimicrobial peptides of the Lynronne family.

Alexis Reinbold^1^, Maxime Gâchon^2,3^, Jean-François Cavalier^4^, Laure Genet^2,3^, Sophie Guillier^2,3^, Mathilde Lescat^2,3^, Muriel Serradeil-Albalat^5^, Linda B. Oyama^6^, Cendrine Nicoletti^1^, Sharon A. Huws^6^, Nicolas Vidal^7^, Fabiano Cavalcanti Fernandes^8^, Andreia Gomes Martins^4^, Stéphane Canaan^4^, Octávio Luiz Franco^9,10^, Sophie Tronnet^1,*^, Olga Iranzo^1,*^, Marc Maresca^1,*^

*^1^	Aix Marseille Univ, CNRS, Centrale Med, iSm2, 13013, Marseille, France; alexis.reinbold51@gmail.com (A.R.), cendrine.nicoletti@univ-amu.fr (C.N.), sophie.tronnet@univ-amu.fr (S.T.), olga.iranzo@univ-amu.fr (O.I.), m.maresca@univ-amu.fr (M.M.)*

*^2^	Unité Bactériologie, Département Microbiologie et Maladies Infectieuses, UMR-MD1-Inserm 1261, Institut de Recherche Biomédicale des Armées (IRBA) - Brétigny-Sur-Orge, 91220, France.*

*^3^ Aix Marseille Univ, INSERM, SSA, MCT, F-13385 Marseille, France. maxime.gachon@intradef.gouv.fr (M.G.), laure.genet@intradef.gouv.fr (L.G.), sophie.guillier@intradef.gouv.fr (S.G), mathilde.lescat@intradef.gouv.fr (M.L)*

*^4^	Aix-Marseille Univ, CNRS, LISM UMR7255, Marseille, France; agomes@imm.cnrs.fr (A.G.M.), canaan@imm.cnrs.fr (S.C.), jfcavalier@imm.cnrs.fr (J.-F.C)*

*^5^	Aix Marseille Univ, CNRS, Centrale Med, FSCM, 13013, Marseille, France; muriel.serradeil@univ-amu.fr (M.S.)*

*^6^ Institute for Global Food Security, School of Biological Sciences, Queen’s University Belfast, Belfast BT9 5DL, UK; s.huws@qub.ac.uk (S.A.H.), l.oyama@qub.ac.uk (L.B.O.)*

*^7^	Yelen Analytics, Yelen-Analytics 10 bd Tempête 13820 Ensues La Redonne, France; yeleniko@yahoo.fr (N.V.)*

*^8^	Departamento de Ciência da Compuagomes@ação, Instituto Federal de Brasília, Campus Taguatinga, Brasília, Brazil fabianofernandesdf@gmail.com (F.C.F.)*

*^9^	S-Inova Biotech, Graduate Program in Biotechnology, Dom Bosco Catholic University, Campo Grande 79117-900, MS, Brazil; ocfranco@gmail.com (O.L.F.)*

*^10^	Center for Proteomic and Biochemical Analysis, Graduate Program in Genomic Sciences and Biotechnology, Catholic University of Brasília, Brasília 71966-700, DF, Brazil; ocfranco@gmail.com (O.L.F.)*

*^*^	Corresponding authors: sophie.tronnet@univ-amu.fr (S.T.), olga.iranzo@univ-amu.fr (O.I.), m.maresca@univ-amu.fr (M.M.)*

# Supplementary data

Analytical RP-HPLC analysis and positive electrospray ionization mass spectrum of the peptide after preparative RP-HPLC purification.

Peptides were analyzed by analytical reversed-phase high-performance liquid chromatography (RP-HPLC) on an Phenomenex Aeris XB-C18 column (250 × 4.6 mm) using a linear gradient of increasing acetonitrile concentration (20-60% B) over 20 min at a flow rate of 1 mL/min (Solvent A: 99.9% water and 0.1% TFA(v/v); Solvent B: 90% acetonitrile, 99.9% water, and 0.1% TFA (v/v)). Elution was monitored by UV absorbance at 220 nm and 280 nm.. The molecular masses of the peptides were determined using a SYNAPT G2 HDMS mass spectrometer (Waters) equipped with a pneumatically assisted atmospheric pressure ionization (API) source. The sample was ionized in positive electrospray mode under the following conditions: electrospray voltage, 2.8 kV; orifice voltage, 20 V; nebulizer gas (nitrogen) flow rate, 100 L/h. The mass spectrum (MS) was obtained using a time-of-flight (TOF) analyzer. ESI-MS analyses were performed at the Spectropole facility (Aix-Marseille University, Marseille, France; <https://fr-chimie.univ-amu.fr/spectropole/>).

**General note for ESI-MS spectra.**

Signals marked with black circles correspond to ions also present in the analytical blank mass spectrum and are therefore not specific to the peptide sample. Peaks marked with green dots correspond to trifluoroacetate (TFA) adduct ions, attributed to residual TFA associated with the peptides after purification. Where multiple TFA adducts are observed, n denotes the number of associated TFA molecules. Zn-containing adducts likely arise from trace Zn present in the ESI-MS system, as peptides containing histidine and/or cysteine residues can coordinate Zn^2+^ under the analysis conditions. Assigned Zn-containing adducts are labelled in the corresponding spectra.

**Figure S1. Analytical RP-HPLC chromatogram (upper) and ESI-MS spectrum (lower) of peptide Lynronne-1.**

**Figure S2. Analytical RP-HPLC chromatogram (upper) and ESI-MS spectrum (lower) of peptide Lynronne-1R.**

**Figure S3. Analytical RP-HPLC chromatogram (upper) and ESI-MS spectrum (lower) of peptide Lynronne-1K.**

**Figure S4. Analytical RP-HPLC chromatogram (upper) and ESI-MS spectrum (lower) of peptide Lynronne-1Inv.**

**Figure S5. Analytical RP-HPLC chromatogram (upper) and ESI-MS spectrum (lower) of peptide Lynronne-1H.**

**Figure S6. Analytical RP-HPLC chromatogram (upper) and ESI-MS spectrum (lower) of peptide Lynronne-2.**

**Figure S7. Analytical RP-HPLC chromatogram (upper) and ESI-MS spectrum (lower) of peptide Lynronne-2R.**

**Figure S8. Analytical RP-HPLC chromatogram (upper) and ESI-MS spectrum (lower) of peptide Lynronne-2K.**

**Figure S9. Analytical RP-HPLC chromatogram (upper) and ESI-MS spectrum (lower) of peptide Lynronne-2Inv.**

**Figure S10. Analytical RP-HPLC chromatogram (upper) and ESI-MS spectrum (lower) of peptide Lynronne-2H.**

**Figure S11. Analytical RP-HPLC chromatogram (upper) and ESI-MS spectrum (lower) of peptide Lynronne-3.**

**Figure S12. Analytical RP-HPLC chromatogram (upper) and ESI-MS spectrum (lower) of peptide Lynronne-3K**

**Figure S13. Analytical RP-HPLC chromatogram (upper) and ESI-MS spectrum (lower) of peptide Lynronne-3H.**

**Figure S14. Concentration–response curves for the cytotoxicity of Lynronnes peptides.** In this figure, L1, L2, and L3 denote Lynronne-1, Lynronne-2, and Lynronne-3, respectively; derivative labels follow the same convention (e.g., L1R denotes Lynronne-1R). Cell viability is expressed as percentage of untreated control / percentage viability. Data are presented as mean ± SEM from n = 3 independent experiments. Curves were fitted using nonlinear regression in GraphPad Prism 11.1.0.

**Figure S15. Concentration–response curves for the haemolytic activity of Lynronnes peptides against human erythrocytes.** In this figure, L1, L2, and L3 denote Lynronne-1, Lynronne-2, and Lynronne-3, respectively; derivative labels follow the same convention (e.g., L1R denotes Lynronne-1R). Haemolysis was quantified as a percentage relative to the positive lysis control. Data are presented as mean ± SEM from n = 3 independent experiments. Curves were fitted using nonlinear regression in GraphPad Prism 11.1.0.

**Table S1 Antibacterial activity of the Lynronnes derivatives against ESKAPEE bacteria. MIC values are expressed in µg/mL.** In this table, L1, L2, and L3 denote Lynronne-1, Lynronne-2, and Lynronne-3, respectively; derivative labels follow the same convention (e.g., L1R denotes Lynronne-1R). Assay performed in n=3.

| **Peptides** | **Gram +** | | | **Gram -** | | | | |
| --- | --- | --- | --- | --- | --- | --- | --- | --- |
|  | ***E. faecalis***  (ATCC 29212) | ***E. faecium***  (ATCC 19434) | ***S. aureus***  (ATCC 6538) | ***A. baumannii***  (ATCC 19606) | ***E. cloacae***  (ATCC 13047) | ***E. coli***  (ATCC 8739) | ***K. pneumoniae***  (ATCC 700603) | ***P. aeruginosa***  (ATCC 9027) |
| L1 | 62.50 | 31.25 | 7.81 | 3.91 | 62.50 | 3.91 | 3.91 | 7.81 |
| L1R | 31.25 | 31.25 | 15.62 | 7.81 | 31.25 | 3.91 | 3.91 | 15.62 |
| L1K | 125 | 125 | 7.81 | 3.91 | 62.50 | 1.95 | 3.91 | 7.81 |
| L1Inv | 62.50 | 31.25 | 7.81 | 3.91 | 31.25 | 1.95 | 1.95 | 7.81 |
| L1H | >500 | >500 | >500 | >500 | >500 | >500 | >500 | >500 |
| L2 | >500 | >500 | 125 | 3.91 | 250 | 7.81 | 3.91 | 7.81 |
| L2R | 250 | 250 | 31.25 | 3.91 | 250 | 3.91 | 1.95 | 7.81 |
| L2K | >500 | >500 | 500 | 7.81 | >500 | 0.98 | 31.25 | 3.91 |
| L2Inv | >500 | >500 | 500 | 3.91 | >500 | 1.95 | 3.91 | 15.62 |
| L2H | >500 | >500 | >500 | >500 | >500 | >500 | >500 | >500 |
| L3 | 62.50 | 31.25 | 62.50 | 31.25 | 125 | 31.25 | 31.25 | 62.50 |
| L3K | 62.50 | 62.50 | 62.50 | 15.62 | 250 | 31.25 | 31.25 | 62.50 |
| L3H | >500 | >500 | >500 | >500 | >500 | >500 | >500 | >500 |
| Amoxicillin | 0.49 | 0.24 | <0.12 | 250 | >500 | 3.91 | >500 | 250 |
| Tetracycline | 31.25 | 0.24 | 0.24 | 3.91 | 0.98 | 0.98 | 31.25 | 62.50 |

**Table S2 Antibacterial activity of the Lynronnes derivatives against E. coli pathogenic and resistant E. coli strains. MIC values are expressed in µg/mL.** In this table, L1, L2, and L3 denote Lynronne-1, Lynronne-2, and Lynronne-3, respectively; derivative labels follow the same convention (e.g., L1R denotes Lynronne-1R). Assay performed in n=3.

| **Peptides** |  | | | | |
| --- | --- | --- | --- | --- | --- |
|  | **(ATCC 8739)** | **EPEC** | **EHEC** | **LF82 AIEC** | **CF1702** |
| L1 | 3.91 | 3.91 | 3.91 | 3.91 | 3.91 |
| L1R | 3.91 | 3.91 | 7.81 | 7.81 | 3.91 |
| L1K | 1.95 | 0.98 | 0.98 | 0.98 | 0.98 |
| L1Inv | 1.95 | 0.98 | 3.91 | 3.91 | 0.98 |
| L1H | >500 | >500 | >500 | >500 | >500 |
| L2 | 7.81 | 3.91 | 3.91 | 3.91 | 1.95 |
| L2R | 3.91 | 3.91 | 3.91 | 3.91 | 1.95 |
| L2K | 0.98 | 3.91 | 1.95 | 0.98 | 0.98 |
| L2Inv | 1.95 | 7.81 | 7.81 | 1.95 | 3.91 |
| L2H | >500 | >500 | >500 | >500 | >500 |
| L3 | 31.25 | 15.62 | 62.50 | 62.50 | 15.62 |
| L3K | 31.25 | 3.91 | 15.62 | 15.62 | 3.91 |
| L3H | >500 | >500 | >500 | >500 | >500 |
| Amoxicillin | 3.91 | 3.91 | 3.91 | >500 | 62.50 |
| Tetracycline | 0.98 | 3.91 | 0.98 | 7.81 | 3.91 |

**Table S3 Antibacterial activity of the Lynronnes derivatives against selected biothreat agents. MIC values are expressed in µg/mL.** In this table, L1, L2, and L3 denote Lynronne-1, Lynronne-2, and Lynronne-3, respectively; derivative labels follow the same convention (e.g., L1R denotes Lynronne-1R). Assay performed in n=3.

| **Peptides** | **Gram +** | **Gram -** | | | | |
| --- | --- | --- | --- | --- | --- | --- |
|  | ***B. anthracis***  (Ames) | ***B. mallei***  (ATCC 23344) | ***B. pseudomallei***  (K96243) | ***B. melitensis***  (NR-256) | ***F. tularensis***  (NR-643) | ***Y. pestis***  (NR-641) |
| L1 | 31.25 | >250 | >250 | 125 (125) | >250 | >250 |
| L1R | 31.25 | >250 | >250 | 62.5 (62.5) | 250 (250) | 250 |
| L1K | 31.25 | >250 | >250 | 250 (250) | >250 | >250 |
| L1Inv | 31.25 | >250 | >250 | 125 (>250) | >250 | >250 |
| L1H | >250 | >250 | >250 | >250 | >250 | >250 |
| L2 | 125 | >250 | >250 | >250 | >250 | >250 |
| L2R | 62.50 | >250 | >250 | >250 | >250 | >250 |
| L2K | >250 | >250 | >250 | >250 | >250 | >250 |
| L2Inv | >250 | >250 | >250 | >250 | >250 | >250 |
| L2H | >250 | >250 | >250 | >250 | >250 | >250 |
| L3 | 125 | >250 | >250 | 250 | 125 (250) | 250 |
| L3K | 125 | >250 | >250 | >250 | 125 (250) | 250 |
| L3H | >250 | >250 | >250 | >250 | >250 | >250 |
| Ciprofloxacin | 0.031 | 1 | 1 | 0.50 | 0.016 | 0.008 |

**Table S4 Antibacterial activity of the Lynronnes derivatives against mycobacteria. MIC values are expressed in µg/mL.** In this table, L1, L2, and L3 denote Lynronne-1, Lynronne-2, and Lynronne-3, respectively; derivative labels follow the same convention (e.g., L1R denotes Lynronne-1R). Assay performed in n=3.

| **Peptides** | ***M. abscessus (S)*** | ***M. abscessus (R)*** | ***M. tuberculosis mc^2^6230*** | ***M. marinum*** |
| --- | --- | --- | --- | --- |
| L1 | 604 ±41 | 516 ±73 | 291 ±19 | >1000 |
| L1R | 754 ±128 | 686 ±73 | 179 ±3 | >1000 |
| L1K | >1000 | 717 ±20.5 | 591 ±13 | 552 ±52 |
| L1I | >1000 | >1000 | 460 ±48 | >1000 |
| L1H | >1000 | >1000 | >1000 | >1000 |
| L2 | >1000 | >1000 | 357 ±12 | 473 ±46 |
| L2R | >1000 | >1000 | 427 ±15 | >1000 |
| L2K | >1000 | >1000 | >1000 | >1000 |
| L2I | >1000 | >1000 | >1000 | >1000 |
| L2H | >1000 | >1000 | >1000 | >1000 |
| L3 | >1000 | >1000 | >1000 | >1000 |
| L3K | >1000 | >1000 | 609 ±51 | >1000 |
| L3H | >1000 | >1000 | >1000 | >1000 |
| Amikacin | 3.4 ±0.1 | 5.9 ±0.3 | 0.37 ±0.02 | 1.7 ±0.04 |
| Isoniazid | *not active* | *not active* | 0.11 ±0.005 | 9.2 ±0.50 |

**Table S5 Effect of salt on the antibacterial activity of the Lynronnes derivatives against E. coli (ATCC 8739) and S. aureus (ATCC 6538). MICs were measured in the presence of physiological salt concentrations (NaCl, 150 mM; MgCl2, 1 mM; CaCl2, 2 mM). MIC are expressed in µg/mL.** In this table, L1, L2, and L3 denote Lynronne-1, Lynronne-2, and Lynronne-3, respectively; derivative labels follow the same convention (e.g., L1R denotes Lynronne-1R). Assay performed in n=3.

| **Peptides** | ***S. aureus* (ATCC 6538)** | | | | ***E. coli* (ATCC 8739)** | | | |
| --- | --- | --- | --- | --- | --- | --- | --- | --- |
|  | **MH** | **MH +**  **NaCl** | **MH +**  **MgCl₂ + CaCl₂** | **MH +**  **NaCl + MgCl₂ + CaCl₂** | **MH** | **MH +**  **NaCl** | **MH +**  **MgCl₂ + CaCl₂** | **MH +**  **NaCl + MgCl₂ + CaCl₂** |
| L1 | 7.81 | 15.62 | 31.25 | 62.50 | 3.91 | 3.91 | 31.25 | 125 |
| L1R | 15.62 | 7.81 | 15.62 | 7.81 | 3.91 | 3.91 | 31.25 | 31.25 |
| L1K | 7.81 | 31.25 | 31.25 | 250 | 1.95 | 3.91 | 62.50 | 250 |
| L1Inv | 7.81 | 15.62 | 15.62 | 62.50 | 1.95 | 3.91 | 31.25 | 125 |
| L1H | >500 | >500 | >500 | >500 | >500 | >500 | >500 | >500 |
| L2 | 125 | 500 | 250 | >500 | 7.81 | 7.81 | 62.5 | 250 |
| L2R | 31.25 | 125 | 62.50 | 500 | 3.91 | 3.91 | 31.25 | 125 |
| L2K | 500 | >500 | >500 | >500 | 0.98 | 7.81 | 125 | 500 |
| L2Inv | 500 | >500 | >500 | >500 | 1.95 | 31.25 | 250 | >500 |
| L2H | >500 | >500 | >500 | >500 | >500 | >500 | >500 | >500 |
| L3 | 62.50 | 125 | >500 | >500 | 31.25 | 31.25 | 62.50 | 125 |
| L3K | 62.50 | 125 | 125 | >500 | 31.25 | 15.62 | 62.50 | 125 |
| L3H | >500 | >500 | >500 | >500 | >500 | >500 | >500 | >500 |
| Amoxicillin | <0.12 | 1.95 | 0.12 | 0.12 | 3.91 | 1.95 | 3.91 | 3.91 |
| Tetracycline | 0.24 | 1.95 | 1.95 | 0.98 | 0.98 | 1.95 | 3.91 | 3.91 |

**Table S6 Antibacterial activities of the Lynronnes derivatives against E. coli (ATCC 8739) and S. aureus (ATCC 6538) in the presence of human serum (50%, 4h incubation).** In this table, L1, L2, and L3 denote Lynronne-1, Lynronne-2, and Lynronne-3, respectively; derivative labels follow the same convention (e.g., L1R denotes Lynronne-1R). MIC are expressed in µg/mL.

| **Peptides** | ***S. aureus* (ATCC 6538)** | | ***E. coli* (ATCC 8739)** | |
| --- | --- | --- | --- | --- |
|  | **MH** | **MH + 50% human serum** | **MH** | **MH + 50% human serum** |
| L1 | 7.81 | 7.81 | 3.91 | 7.81 |
| L1R | 15.62 | 7.81 | 3.91 | 3.91 |
| L1K | 7.81 | 7.81 | 1.95 | 0.98 |
| L1Inv | 7.81 | 7.81 | 1.95 | 3.91 |
| L1H | >500 | >500 | >500 | >500 |
| L2 | 125 | 500 | 7.81 | 3.91 |
| L2R | 31.25 | >500 | 3.91 | 7.81 |
| L2K | 500 | >500 | 0.98 | 1.95 |
| L2Inv | 500 | >500 | 1.95 | 3.91 |
| L2H | >500 | >500 | >500 | >500 |
| L3 | 62.50 | 125 | 31.25 | 125 |
| L3K | 62.50 | 15.62 | 31.25 | 15.62 |
| L3H | >500 | >500 | >500 | >500 |
